# Mechanisms of Action in a Behavioral Weight-Management Program: Latent Growth Curve Analysis

**DOI:** 10.1093/abm/kaab019

**Published:** 2021-04-03

**Authors:** Sarah Bates, Paul Norman, Penny Breeze, Alan Brennan, Amy L Ahern

**Affiliations:** 1 School of Health and Related Research, University of Sheffield, Sheffield, UK; 2 Department of Psychology, University of Sheffield, Sheffield, UK; 3 MRC Epidemiology Unit, University of Cambridge, Cambridge, UK

**Keywords:** Weight management, Mediation, Restraint, Habit, Self-regulation

## Abstract

**Background:**

A greater understanding of the mechanisms of action of weight-management interventions is needed to inform the design of effective interventions.

**Purpose:**

To investigate whether dietary restraint, habit strength, or diet self-regulation mediated the impact of a behavioral weight-management intervention on weight loss and weight loss maintenance.

**Methods:**

Latent growth curve analysis (LGCA) was conducted on trial data in which adults (*N* = 1,267) with a body mass index (BMI) ≥28 kg/m^2^ were randomized to either a brief intervention (booklet on losing weight), a 12 week weight-management program or the same program for 52 weeks. LGCA estimated the trajectory of the variables over four time points (baseline and 3, 12 and 24 months) to assess whether potential mechanisms of action mediated the impact of the weight-management program on BMI.

**Results:**

Participants randomized to the 12 and 52 week programs had a significantly greater decrease in BMI than the brief intervention. This direct effect became nonsignificant when dietary restraint, habit strength, and autonomous diet self-regulation were controlled for. The total indirect effect was significant for both the 12 (estimate = −1.33, standard error [*SE*] = 0.41, *p* = .001) and 52 week (estimate = −2.13, *SE* = 0.52, *p* < .001) program. Only the individual indirect effect for dietary restraint was significant for the 12 week intervention, whereas all three indirect effects were significant for the 52 week intervention.

**Conclusions:**

Behavior change techniques that target dietary restraint, habit strength, and autonomous diet self-regulation should be considered when designing weight loss and weight loss maintenance interventions. Longer interventions may need to target both deliberative and automatic control processes to support successful weight management.

## Teaser

The effect of a behavioural weight-management intervention on weight loss was explained by changes in dietary restraint, habit strength and autonomous motivation

## Graphical Abstract

**Figure:**
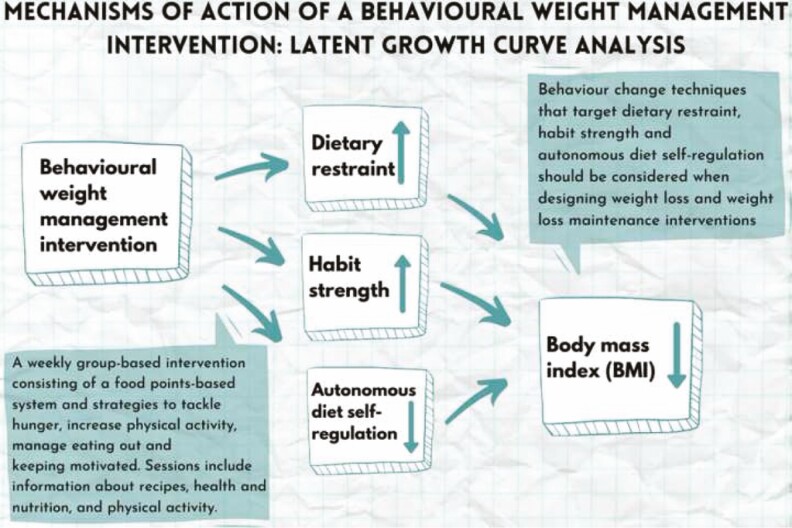

## Introduction

Approximately two thirds of adults in the UK and USA are classed as being overweight or obese based on their body mass index (BMI) and there is little evidence that the prevalence is decreasing [1, 2]. Behavioral weight-management programs are the first-line treatment for people classed as overweight or obese [3] and, although there is evidence that these are effective [4], the results are heterogeneous between and within studies [5]. In a systematic review, the average weight loss across randomized controlled trials (RCTs) of nonsurgical weight loss interventions varied from −4.03 to −21.3 kg [6]. There is also variability in evidence for the duration of the intervention effect. A systematic review of trials with a follow-up of at least 16 weeks found evidence for significant intervention effects ranging from 18 months to 5 years from baseline [7].

The heterogeneity in the size and duration of treatment effect may be due to differences in the behavior change techniques (BCTs) used in an intervention and the mechanisms of actions targeted. For example, in a previous trial, an intervention that used BCTs, such as developing implementation intentions to target habit formation, resulted in greater weight loss than an intervention that used BCTs, such as education about misinformation to target unhealthy relationships with food [8]. Given the similarities in the duration and mode of delivery, the findings indicate that the different BCTs used, and mechanisms of action targeted, resulted in differences in weight change. Identifying relevant mechanisms of action associated with the desired outcome will enable the evidence-based selection of BCTs to include in an intervention [9]. This is particularly important for weight loss maintenance as weight regained postintervention is commonly reported (e.g., [7]). Thus, a greater understanding of the mechanisms of action associated with short- and longer-term weight loss is needed to inform the design of effective interventions, through the selection of appropriate BCTs, that result in both weight loss and weight loss maintenance.

There are many potential mechanisms of action for weight-management interventions. A common focus of these interventions is to create healthy eating behaviors by restricting the amount and types of food and drinks consumed [10]. Efforts to restrict food intake, such as using strategies to prevent overeating (e.g., portion control or avoiding unhealthy foods), adjusting eating behavior after over consuming, and being conscious of food choices in order to control weight are often referred to as dietary restraint [11]. A recent review of studies that measured dietary restraint found that restraint was associated with weight loss [12]; specifically, higher dietary restraint was associated with a lower weight in populations with obesity, and increases in dietary restraint were associated with greater weight loss. In studies that have examined weight loss maintenance, increases in dietary restraint during weight loss have also been found to predict weight loss maintenance [13] and decreases in dietary restraint have been found to be associated with greater weight regain over 18 months [14] to 10 years [8]. Although there is evidence from observational and RCTs that changes in dietary restraint are associated with weight control [12, 15], there has been less research on dietary restraint as a mechanism of action (i.e., mediator) of weight-management interventions. In a review, only one study had conducted formal mediation analysis [16], reporting that dietary restraint mediated the impact of a weight-management intervention on weight loss over 24 months [17]. In a more recent study, dietary restraint was not found to mediate the effect of a weight-management intervention; however, the intervention included meal plans and prepackaged food, which may have limited the opportunity for participants to practice restrained eating [18].

Continued dietary restraint may lead to healthy dietary behaviors becoming habitual, which, in turn, may aid the maintenance of weight loss. Habits can be defined as learned stimulus–response associations such that when a stimulus is encountered, an individual responds automatically with a certain behavior or set of behaviors [19, 20]. Habits are formed when a behavior, such as monitoring diet, eating fruit and vegetables, or taking part in physical activity, is repeated frequently in the same context such that a cognitive association is made between the situation and behavior [21]. Habit strength has been associated with eating behaviors in observational studies [22, 23] and decreases in BMI during a weight loss intervention [24]. In addition, in a weight loss maintenance intervention, increases in healthy eating habits were associated with decreases in BMI over 1 year [25]. Although there has been some research on the benefits of habit-based interventions [9, 26], there is little research on whether habit strength is a mechanism of action of effective interventions. In one study, the effect of a brief habit-based weight loss intervention was mediated by automaticity [27]. However, this analysis was conducted over a short time period (3 months) and only one item was used to assess automaticity.

The motivation that drives behavior change is also a key factor in weight loss and weight loss maintenance [28]. Autonomous regulation occurs when engaging in a behavior is autonomously motivated; that is, the behavior is perceived as valued, important to the individual, consistent with intrinsic goals or outcomes, and part of the individual’s identity [29]. It is predicted that those with higher autonomous self-regulation are more likely to adhere to the behavior change desired [30], and this is supported by findings that increased autonomous self-regulation is associated with adherence to self-monitoring behavior [31], weight loss [31, 32], and weight loss maintenance [33]. In contrast, controlled regulation is driven by external pressures, such as a reward or avoidance of negative consequences. Although there is evidence that controlled regulation results in success in the short term [34], it is predicted that without autonomous regulation, positive changes in behaviors and weight loss will not be maintained [28]. In a systematic review of mediators of weight loss [16], only one study examined the mediating role of autonomous self-regulation [35]; an intervention aimed at promoting autonomous regulation resulted in greater weight loss than a general health education program and intervention effects on 3 year weight change were partially mediated by autonomous self-regulation at 2 years [33], supporting the proposition that autonomous diet self-regulation contributes to weight loss maintenance [28].

Overall, although there is evidence that dietary restraint, habit strength, and autonomous self-regulation are associated with weight control, there have been few formal mediation analyses examining whether change in these factors mediate the impact of effective interventions. In addition, of those mediation analyses that have been conducted, traditional regression methods have been used, which only examine two time points. This results in the loss of information or requires several analyses between each set of time points. Using only two time points, especially the start and end of a study means that the model does not represent the trajectory of weight throughout the intervention and follow-up [36]. Latent growth curve analysis (LGCA) enables the analysis of the full trajectory of a variable over time. This is particularly important when individual changes follow a nonlinear trajectory, which is likely in a weight-management intervention in which a greater change during the active intervention than during follow-up is often expected [6]. LGCA also enables variables to be both outcomes and predictors so that the trajectory of a potential mediator can be conditional on demographics factors while also being a predictor of an outcome. This method allows a greater understanding of the complex associations between treatment, mechanisms of action, and outcomes over time [36].

### The Present Study

Secondary mediation analysis was conducted on data from the Weight loss Referrals for Adults in Primary care trial (the weight loss referrals for adults in primary care [WRAP] trial), which examined the effectiveness and cost-effectiveness of a 52 week referral to an open-group behavioral weight-management program (WW, formerly Weight Watchers) compared to a 12 week referral to the same program and a brief intervention (written materials on how to lose weight) [37]. Participants assigned to the 12 and 52week weight-management programs lost significantly more weight than the control group at 3 and 12 months and those assigned to the 52 week program lost significantly more weight than the 12 week program and the brief intervention at 12 and 24 months. The full results are reported in Ahern et al. [37]. The aim of the present study was to investigate whether the trajectories of dietary restraint, habit strength, and autonomous, controlled, and amotivation self-regulation of diet mediated the effect of the weight-management program on BMI trajectory over 24 months using LGCA, a method that incorporates the full trajectory of the mediators and BMI.

## Method

### Participants

Eligible participants were aged 18 years or older with a BMI of 28 kg/m^2^ or above and were recruited through general practice records in England. Eligible individuals were identified by their primary care providers. Patients who were pregnant or were planning pregnancy within 2 years, had past or planned bariatric surgery, were already participating in a structured monitored weight-management program, were taking part in other research that would impact on the study outcomes, had a diagnosed eating disorder, or were unable to understand study information were excluded. Practices also excluded patients considered ineligible for other reasons not stated above, such as terminal illness or a mental health diagnosis. Eligible participants were then invited to take part in the study by letter and asked to contact a study coordinator for a telephone screening if interested in participating. Eligible and willing participants were given an appointment where weight and height were measured to confirm eligibility. All participants gave written informed consent [37].

### Interventions

Participants were randomly assigned to either a brief intervention, a 12 week referral to an open-group behavioral weight-management program (WW, formerly Weight Watchers) or a 52 week referral to the same program in a 2:5:5 allocation stratified by center and gender using a randomization sequence generated by the trial statistician.

The brief intervention included the recognition of the problem by the GP in the form of a letter and written information on self-help weight loss strategies (British Heart Foundation Booklet: So you want to lose weight…for good). At the baseline visit, participants were read a scripted introduction that drew attention to each section of this booklet. The 12 and 52 week behavioral weight-management programs were group based and led by an individual who had personal experience of successful weight management. It included one-to-one discussions with participants at their first session and during the part of the session when participants were weighed [38]. Sessions were held once a week at community-based venues and were an hour long. The core program material consisted of a food points-based system (calculated based on the participant’s age, gender, height, weight, and activity) and strategies to tackle hunger, increase physical activity, manage eating out, and keeping motivated. Sessions also included information about recipes, health and nutrition, and physical activity. Weight loss goals were between 0.5 and 1 kg per week based on a deficit of 500 kcal per day. Participants were encouraged to be physically active and work toward a goal of 10,000 steps per day. The intervention used food and activity diaries, goal setting, evaluation of progression and the provision of rewards for reaching weight loss targets. Using the taxonomy described by Michie et al. [39], the intervention content has retrospectively been categorized into the following BCTs: provide general information on behavior-health link, prompt intention formation, prompt review of behavioral goals, prompt self-monitoring of behavior, provide feedback on performance, provide contingent rewards, set graded tasks, provide opportunities for social comparison, instruction on how to perform a behavior, information from a credible source (i.e., someone with experience of successful weight management), social support, relapse prevention, and restructuring the food environment [40, 41].

Participants assigned to the behavioral weight-management programs were given vouchers to attend weekly sessions and use online tools for the duration of their intervention. Those allocated to the 12 week referral received vouchers to attend 12 group sessions and access to internet resources for 16 weeks and those allocated to the 52 week referral received vouchers for 52 sessions and access to internet resources for 12 months [42]. The vouchers covered the full cost of the sessions and access to online resources.

### Measures

BMI and potential mediators were collected at baseline and 3, 12 and 24 months.

#### Body mass index

Height was measured at baseline to the nearest 0.1 cm using a stadiometer, and weight was measured to the nearest 0.1 kg using a four-point segmental body composition analyzer at all time points. This was used to calculate BMI (kg/m^2^).

#### Dietary restraint

A 14-item subscale of the Three-Factor Eating Questionnaire [12, 43] was used to assess two types of restraint: rigid control, which refers to an all-or-nothing perception of weight control, and flexible control, which refers to more adaptability in eating behaviors to control weight. In the current study, the two types of restraint were highly correlated (*r* = .89), so the total subscale score was used (alpha = .86). This reflects findings from other studies in which dieting behavior and weight loss are associated with similar increases in both rigid and flexible dietary restraint [44, 45]. The measure includes items such as “I deliberately take small helpings as a measure of weight control.” Eight items have a true/false response option and the remaining six items are presented with a four-point Likert scale. Higher scores on this measure represent greater control over dietary behaviors [11, 43].

#### Self-report habit index

The self-report habit index [46] was used to measure habit strength. The measure includes items assessing behavioral frequency, automaticity, and identity (alpha = .89). The statement “Watching what I eat is something” was followed by 12 items, such as “I do frequently” or “would require effort not to do it.” The items were accompanied by seven-point Likert scales from agree to disagree. Higher scores indicate that the behavior is more habitual.

#### Diet self-regulation

The measure of diet self-regulation was adapted from the treatment self-regulation questionnaire [47] to assess self-regulation of eating a healthy diet. The measure “The reason I would eat a healthy diet is” is followed by 15 items split into three subscales. The autonomous self-regulation subscale (alpha = 0.81) includes six items such as “Because it is consistent with my life goals.” The controlled self-regulation subscale (alpha = 0.88) includes six items such as “Because I want others to approve of me.” The amotivation self-regulation subscale (alpha = 0.79), a measure of the absence of motivation, included three items such as “I do not really think about it.” All items were presented with a seven-point Likert scale from not at all true to very true.

### Statistical Analysis

To examine the longitudinal associations between the potential mediators and BMI, LGCA was conducted. This type of analysis, in which a curve is fitted to the variable at each of the four time points, allows examination of the trajectory of variables over the 2 years. More detail about this analysis method can be found in the Supplementary Material. All analyses were conducted using Mplus8, Version 1.6 [1]. Maximum likelihood estimation was used for all models. The analysis was conducted in three stages.

#### Step 1. Fit a latent growth curve to each variable

**Fig. 1.**
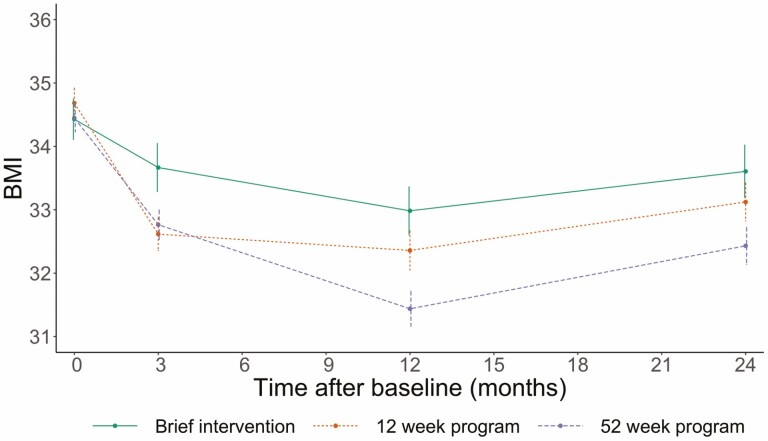
Mean change in BMI in each treatment group over 24 months.

**Fig. 2.**
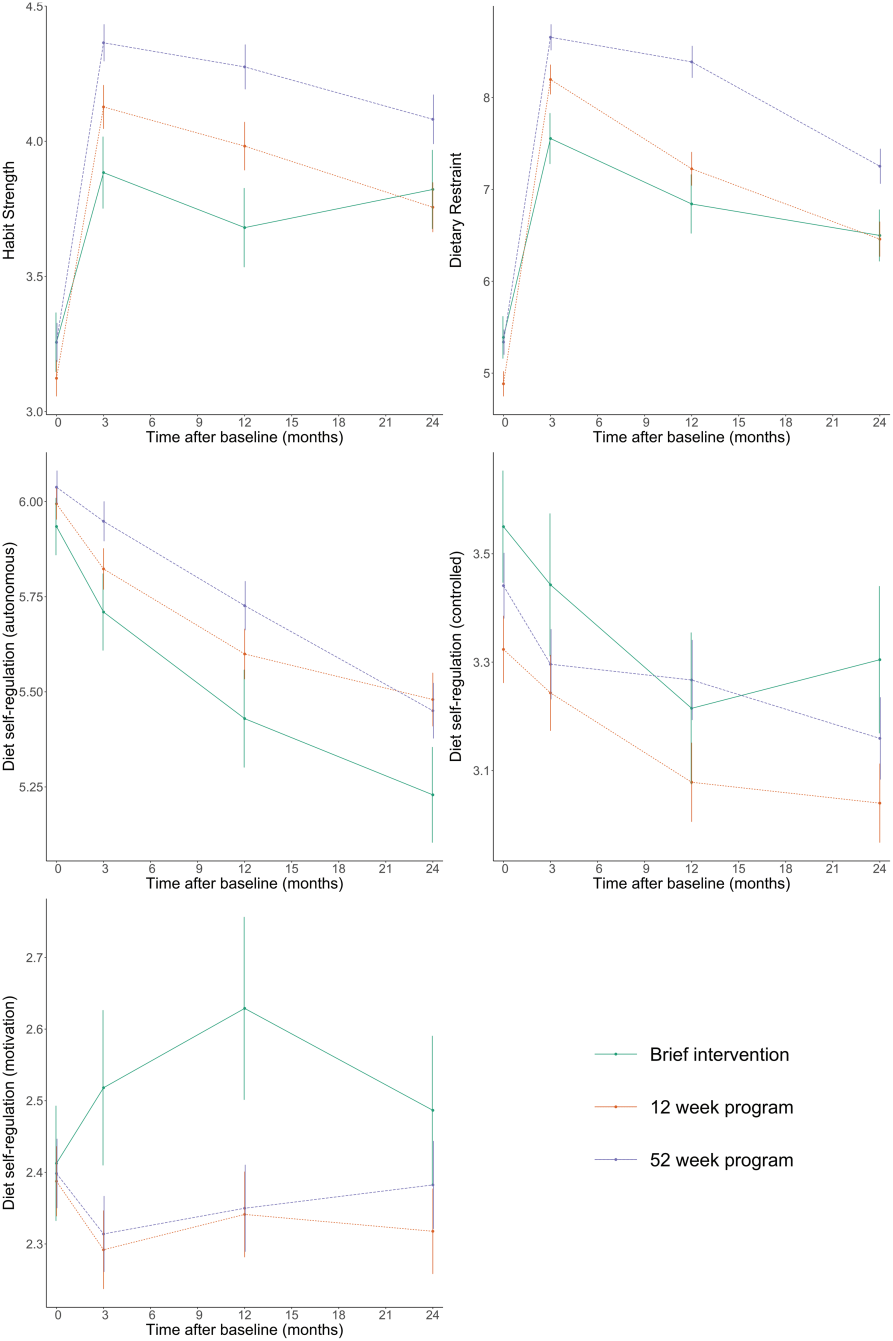
Mean change in habit strength, dietary restraint, and diet self-regulation subscales in each treatment group over 24 months.

Scores at baseline and 3, 12, and 24 months were used to fit a curve to BMI, dietary restraint, habit strength, and the three subscales of diet self-regulation: autonomous, controlled, and amotivation. The intercept factor represented the values at baseline and the slope and quadratic factors represented the change in variables between baseline and 24 months. The means of each variable over the four time points were examined to determine the likely shape of the curve (i.e., linear or quadratic). First, a simple model was fitted in which there was a single growth factor with a variance of zero. Then, as recommended [48], increasingly complex models were fitted and compared. At each stage, if the simpler model had a better or equal fit to the more complex model, it was chosen for analysis. An example of the path diagram for the unconditional model is in Supplementary Fig. 3. Once the best fitting unconditional model was chosen, variables were added to form the conditional model [36]. Age, gender, and treatment group were included as control variables for each latent growth factor. For the BMI curve, income and education were also controlled based on evidence that these demographic factors are associated with BMI [49]. These additional factors were not included in the curve for the potential mediators due to the lack of evidence supporting an association. Path diagrams for the conditional models are in Supplementary Figs. 4 and 5. A piecewise analysis was also fitted, splitting the trajectories of BMI and potential mediators into two latent growth curves based on the initial change (baseline to either 3 or 12 months depending on the trajectory of the variable; Figs. 1 and 2) and the subsequent return toward baseline values. This analysis was conducted to determine whether piecewise models resulted in a better fit to the variables and to explore the relationships between BMI and potential mediators at different time points in the trial.

#### Step 2. Examine associations between change in potential mediator variables and change in BMI

Parallel processes models were developed for each of the potential mediator variables and BMI. These models allow the examination of the correlation between the growth curves fitted in step one. Specifically, the curve fitted to the potential mediators in the previous step was (individually) combined with the curve fitted to the BMI trajectory to determine the correlations between the latent growth factors of the two variables.

#### Step 3. Mediation models

If the trajectory of a potential mediator was associated with group allocation (identified in Step 1) and with the BMI trajectory (Step 2), then it was included in the full mediation model. The curves fitted to the potential mediators and BMI in Step 1 were combined in a single model in which the trajectory of BMI was conditional on the trajectory of potential mediators. The significance of the individual indirect effects of each mediator, total indirect effect, and the direct effect between the intervention and the BMI was examined to determine whether the intervention effect was mediated.

#### Model fit

Model fit was checked at each stage. The criteria used to make a judgment on model fit were a comparative fit index (CFI) above or equal to 0.95, root mean square error of approximation (RMSEA), and standardized root mean-square residual (SRMR) below or equal to 0.08 [36]. A nonsignificant value of the chi-square (χ ^2^) statistic is often used to judge model fit; however, due to the large sample size, which often results in a significant value even with a good model fit [48], this criterion was not used in this study. The fit of each model was assessed using all criteria.

### Missing Data

The percentage of participants who completed the assessments at 3, 12, and 24 months was 79%, 65%, and 68%, respectively. The percentage of missing data for each treatment group and specifically for BMI and the measures are reported in Supplementary Tables 3 and 4. The pattern of missing data was assessed and was treated as missing not at random. There was an increasing number of missing values at later time points and it is probable that dropout was linked to treatment effectiveness [50]. Multiple imputation was conducted using R. For each variable, the missing values were predicted; the variables selected for prediction were based on the strategy outlined by van Buuren et al. [51]. A prediction matrix (Supplementary Fig. 2) shows the variables that were used to predict missing values for each variable. Full details of the method used are in the Supplementary Material. Convergence plots confirmed that convergence had been achieved and strip plots showed that the imputed values did not go out of the range of the actual values and that they followed the same distribution.

## Results

### Baseline Characteristics

Between 18 October 2012 and 10 February 2014, 1954 participants were screened and 1,267 were eligible and were randomly allocated to a condition [37]. The baseline characteristics of the participants (*N* = 1,267) including psychological variables are in Table 1. Additional participant characteristics can be found in the original reporting of the study [37]. The change in both BMI and the psychological/behavioral variables are shown in Figs. 1 and 2. There were no significant differences between the treatment groups at baseline on BMI or the potential mediators determined by one-way analysis of variance tests. BMI and the mediator variables showed change between baseline and 3 or 12 months before a stabilization or return toward baseline between 12 and 24 months. Autonomous diet self-regulation decreased over the 24 months for all intervention groups.

**Table 1.** Baseline characteristics of participants in the weight loss referrals for adults in primary care (WRAP) trial (*N* = 1,267)

	Treatment group					
	Brief intervention		12 week intervention		52 week intervention	
	*n*	%	*n*	%	*n*	%
Gender						
Female	143	68	357	68	358	68
Male	68	32	171	32	170	32
Education						
None	7	3	25	5	27	5
GCSE/A-level/equivalent	108	51	247	47	265	50
University degree or higher/equivalent	81	38	199	38	174	33
Missing	15	7	54	10	60	11
Income						
Under £20,000	65	33	124	25	138	28
£20–£49,999	66	33	173	35	176	35
£50,000+	41	21	91	18	84	17
Prefer not to say or missing	27	13	111	22	100	20
	*M*	*SD*	*M*	*SD*	*M*	*SD*
Age	51.91	14.07	53.60	12.27	53.29	13.98
BMI	34.43	4.63	34.68	5.39	34.45	5.05
Dietary restraint	5.39	3.26	4.88	3.03	5.34	3.06
Habit strength	3.24	1.38	3.08	1.29	3.14	1.38
Diet self-regulation						
Amotivation	2.41	1.14	2.39	1.10	2.40	1.09
Autonomous	5.93	1.07	5.99	0.92	6.04	0.97
Controlled	3.55	1.47	3.32	1.39	3.44	1.36

*BMI* body mass index; *SD* standard deviation; GCSE general certificate of secondary education.

### Latent Growth Curve Analysis

#### Step 1. Fit a latent growth curve to each variable

A latent growth curve was fitted to the four time points (baseline and 3, 12, and 24 months) for BMI, dietary restraint, habit strength, and the three subscales of diet self-regulation (autonomous, controlled, and amotivation). A quadratic growth curve was the best fitting model for all variables other than the amotivation subscale of diet self-regulation for which an intercept-only model was the best fit. For the other four potential mediators (dietary restraint, habit strength, and autonomous and controlled diet self-regulation), the model was able to converge and fitted best when the variance of the quadratic factor was set to 0. The model for BMI fitted well without this restriction. The results from the increasingly complex unconditional models are reported in Supplementary Tables 5–10. Once the best fitting unconditional model was established, the conditional factors were added. The values for each of the latent growth factors along with fit statistics of the conditional model are shown in Table 2. The model fit for all variables was good for all the criteria other than the model for BMI, which did not meet the cutoff criteria for CFI and RMSEA. However, the values were close to the criteria, indicating that the model provided a reasonable description of the data.

**Table 2.** Model fits to trajectory of BMI and psychological/behavioral variables

Variable	Intercept	Slope	Quadratic	CFI	RMSEA	SRMR
BMI	36.16 (1.02)***	0.84 (0.95)	0.03 (0.32)	0.93	0.12	0.02
Dietary restraint	2.82 (0.37)***	2.68 (0.59)***	−0.87 (0.19)***	0.97	0.05	0.04
Habit strength	1.94 (0.19)***	0.79 (0.27)**	−0.21 (0.11)	0.97	0.05	0.04
DSR autonomous	5.75 (0.13)***	−1.16 (0.33)***	0.36 (0.13)**	0.96	0.05	0.02
DSR controlled	3.10 (0.17)***	−0.08 (0.30)	0.09 (0.13)	0.99	0.03	0.02
DSR amotivation	2.39 (0.12)***	NA	NA	0.96	0.04	0.05

*BMI* body mass index; *CFI* comparative fit index; *DSR* diet self-regulation; *RMSEA* root mean square error of approximation; *SRMR* standardized root mean square residual.

*p < .05, **p < .01, ***p < .00.

Table 3 shows the full details of the associations between the latent growth factors of each variable and age, gender, and treatment group in the conditional models. There were significant effects of both the 12 and 52 week program on the slope and quadratic of the BMI trajectory, controlling for age, gender, income, and education. There were significant effects of both the 12 and 52 week program on the slope and quadratic factors of dietary restraint and habit strength but only the 52 week intervention significantly impacted autonomous diet self-regulation. Age and gender were controlled for in all models. Gender was associated with the slope and quadratic of dietary restraint and controlled diet self-regulation, and age was associated with the slope and quadratic of autonomous diet self-regulation.

**Table 3.** Coefficients of age, gender, and group allocation on trajectories of BMI and potential mediators

Variable	Gender (reference group male)	Age	Treatment group (reference brief intervention)	
			12 week group	52 week group
BMI				
Intercept	1.18 (0.31)***	−0.04 (0.01)**		
Slope	−0.52 (0.27)	−0.03 (0.01) **	−0.91 (0.38)**	−1.82 (0.39)***
Quadratic	0.11 (0.10)	0.01 (0.003)	0.37 (0.13)**	0.66 (0.13) ***
Dietary restraint				
Intercept	1.56 (0.18)***	0.02 (0.01)***		
Slope	−0.86 (0.24)**	0.01 (0.01)	0.87 (0.29)**	1.50 (0.31)***
Quadratic	0.23 (0.08)**	−0.003 (0.003)***	−0.30 (0.10)**	−0.47 (0.10)***
Habit				
Intercept	0.24 (0.09)**	0.02 (0.003)***		
Slope	0.06 (0.11)	−0.004 (0.004)	0.36 (0.14)*	0.57 (0.14)***
Quadratic	−0.03 (0.04)	0.002 (0.002)	−0.16 (0.06)*	−0.23 (0.06)***
Diet self-regulation autonomous				
Intercept	0.27 (0.06)***	−0.001 (0.002)		
Slope	−0.24 (0.13)	0.01 (0.01)*	0.21 (0.16)	0.40 (0.17)*
Quadratic	0.09 (0.05)	−0.01 (0.002)*	−0.06 (0.07)	−0.15 (0.07)*
Diet self-regulation controlled				
Intercept	0.17 (0.08)*	0.003 (0.003)		
Slope	−0.38 (0.13)**	−0.001 (0.01)	0.12 (0.13)	0.29 (0.17)
Quadratic	0.12 (0.05)*	−0.001 (0.002)	−0.05 (0.06)	−0.12 (0.07)
Diet self-regulation amotivation				
Intercept	−0.07 (0.03)	0.02 (0.04)*		

*BMI* body mass index.

*p < .05, **p < .01, ***p < .001.

There were significant associations between the BMI intercept and slope (estimate = −2.31, standard error [*SE*] = 0.77, *p* = .002), intercept and quadratic (estimate = 0.72, *SE* = 0.30, *p* = .02), and slope and quadratic growth factors (estimate = −2.81, *SE* = 0.30, *p* < .001), indicating that a higher BMI at baseline was associated with a steeper decline in BMI and a steeper return toward the baseline BMI. There were also significant correlations between the intercepts and slopes of dietary restraint (estimate = 0.40, *SE* = 0.12, *p* = .001) and controlled diet self-regulation (estimate = −0.05, *SE* = 0.02, *p* = .02), indicating that higher baseline values resulted in a lower slope (lesser increase) for controlled diet self-regulation and a higher slope (greater increase) for dietary restraint. The correlations between the intercept and slope of autonomous diet regulation (estimate = 0.03, *SE* = 0.02, *p* = .10) and habit strength (estimate = -.04, *SE* = 0.05, *p* = 0.41) were nonsignificant. Piecewise latent growth curves were fitted to the trajectories of BMI and the potential mediators; however, this resulted in a poorer fit than the quadratic model. Full results are in the Supplementary Tables 11–14.

#### Step 2. Examine associations between change in potential mediator variables and change in BMI

The associations between each of the latent growth factors of the potential mediator variables and the latent growth factors of BMI along with the model fit statistics are in Table 4. There were negative associations between the slopes of BMI and three potential mediator variables; dietary restraint (estimate = −0.60, *SE* = 0.20, *p* = .003), habit strength (estimate = −0.36, *SE =* 0.08, *p* < .001), and autonomous diet self-regulation (estimate = −0.87, *SE* = 0.25, *p* < .001). Increases in these potential mediators were associated with decreases in BMI. At baseline, a higher controlled diet self-regulation score was associated with a higher BMI (estimate = 0.71, *SE* = 0.19, *p* < .001) but the association between the slopes was nonsignificant (estimate = −0.02, *SE* = 0.06, *p* = .74). The amotivation subscale of diet self-regulation was specified as an intercept-only model, so the correlation of the change over time in this variable with change in BMI could not be examined. Although the curve of the potential mediator variables were quadratic, the quadratic growth factors were fixed to 0 and, therefore, the correlation between this and the BMI growth factors could not be calculated. Although three models fell slightly below the criteria recommended for the CFI, all were close and met other measures of fit.

**Table 4.** Correlations between the latent growth factors of BMI and potential mediators

Variable	BMI growth factors			Fit statistics		
	Intercept	Slope	Quadratic	CFI	RMSEA	SRMR
Dietary restraint						
Intercept	−0.57 (0.36)	−0.28 (0.36)	0.10 (0.12)	0.94	0.08	0.03
Slope	0.20 (0.22)	−0.60 (0.20)**	0.11 (0.07)			
Habit strength						
Intercept	−0.35 (0.19)	0.05 (0.17)	−0.01 (0.06)	0.95	0.08	0.03
Slope	0.12 (0.10)	−0.36 (0.08)***	0.08 (0.03)**			
Autonomous diet self-regulation						
Intercept	0.22 (0.14)	0.10 (0.11)	−0.03 (0.04)	0.93	0.08	0.06
Slope	−0.45 (0.31)	−0.87 (0.25)***	0.25 (0.09)**			
Controlled diet self-regulation						
Intercept	0.71 (0.19)***	−0.03 (0.15)	0.02 (0.05)	0.94	0.07	0.02
Slope	−0.12 (0.08)	−0.02 (0.06)	−0.001 (0.02)			

*BMI* body mass index; *CFI* comparative fit index; *RMSEA* root mean square error of approximation; *SRMR* standardized root mean square residual.

*p < .05, **p < .01, ***p < .001.

In the piecewise analyses, associations between the slopes of the mediators in the intervention (0–12 months) and maintenance phases (12–24 months) were examined. In the intervention phase, the BMI slope was associated with the slopes of dietary restraint, habit strength, and autonomous diet self-regulation. The BMI slope in the maintenance phase was associated with the slope of autonomous diet self-regulation in the intervention phase and the slope of habit in the maintenance phase. However, the fit of the piecewise models was poor based on model fit statistics (Supplementary Table 15). Therefore, these results should be interpreted with caution and a full mediation model was not examined.

#### Step 3. Mediation models

In Step 1, it was determined that there were treatment effects of both the 12 and 52 week intervention on BMI trajectory compared to the control group. Of the potential mediators, dietary restraint, habit strength, and autonomous diet self-regulation were associated with both treatment group (Step 1) and BMI trajectory (Step 2). The amotivation and controlled subscales of diet self-regulation did not fit these criteria and, therefore, were not included.

**Fig. 3.**
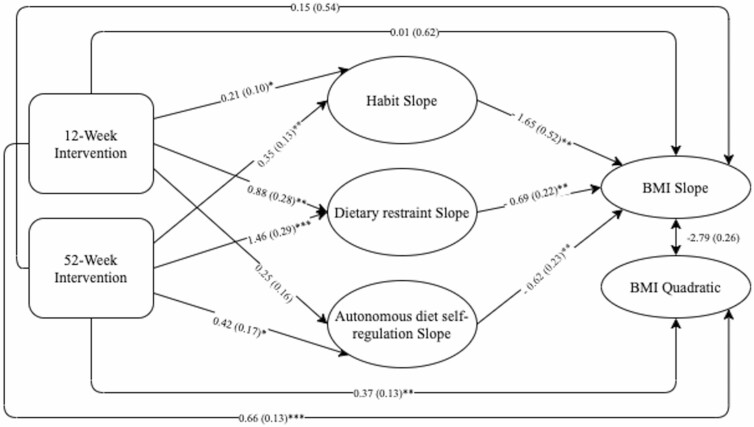
Mediation path diagram.

Mediation models were tested to determine whether the impact of the intervention on BMI slope was mediated by the slope of dietary restraint, habit strength, and autonomous diet self-regulation (the variance of the quadratic variables was restricted to 0 and, therefore, could not be included as a mediator). The results of the separate models for each of the potential mediators are in Supplementary Table 16 and indicate that dietary restraint and habit strength were significant mediators of the 12 week intervention and that all three variables were significant mediators of the 52 week intervention. A full mediation model with all three mechanisms of action was then tested. When fitted, the total effects of both interventions on BMI slope were significant and the direct effects became nonsignificant (Table 5). The total indirect effect via the three mediator variables was significant; for the 12 week intervention effect, only the individual indirect effect of dietary restraint was statistically significant, whereas for the 52 week intervention, the individual indirect of all three variables were significant. Effect sizes were larger for the 52 week program than the 12 week program on all mediators but only significantly larger for dietary restraint and habit strength. Model fit statistics indicate an adequate fit on RMSEA (0.06) and SRMR (0.06) measures and was close to the fit criteria for CFI (0.94). The results of this are shown in Table 5 and a simplified model is included in Fig. 3 (full model tested is presented in Supplementary Fig. 6).

**Table 5.** Total, direct, and indirect effects via mediating variables of the 12 and 52 week intervention on BMI

Effects	12 week intervention			52 week intervention		
	Estimate	SE	*p*	Estimate	SE	*p*
Total impact of intervention on BMI	−0.69	0.36	.04	−1.72	0.38	<.001
Direct effect of intervention on BMI when mediators included	0.64	0.54	.23	0.42	0.63	.51
Total indirect effect of mediating variables	−1.33	0.41	.001	−2.13	0.52	<.001
Indirect effect of mediators						
Dietary restraint	−0.61	0.27	.02	−0.98	0.39	.008
Habit strength	−0.56	0.29	.06	−0.88	0.25	.018
Autonomous diet self-regulation	−0.17	0.54	.23	−0.27	0.62	.048

*BMI* body mass index; *SE* standard error.

## Discussion

Dietary restraint, habit strength, and autonomous diet self-regulation mediated the effect of a weight-management program on BMI change. The 12 and 52 week programs were both associated with increases in dietary restraint and habit strength and the 52 week program was also associated with a lower reduction in autonomous diet self-regulation. These changes were associated with decreases in BMI over the 2 years. When controlling for change in habit strength, dietary restraint, and autonomous diet self-regulation, the impact of both the 12 and 52 week programs on the slope of BMI became nonsignificant. Although the combined indirect effect was significant for both the 12 and 52 week interventions, for the shorter intervention, only the individual direct effect of dietary restraint was significant, whereas the indirect direct effect of all three variables were significant for the 52 week intervention.

This intervention included several BCTs and so it is not possible to establish which specific BCTs or combination of BCTs resulted in the increases in dietary restraint and habit strength observed during the 12 and 52 week weight-management programs. However, the intervention included several BCTs that have been linked with behavioral regulation, including self-monitoring of behavior and outcomes, through food and activity diaries and regular weight measurement, goal setting, and action planning [52, 53]. Behavioral regulation is defined as behavioral, cognitive, and/or emotional skills for managing or changing behavior [52, 53]. Given that dietary restraint can be considered as behavioral and cognitive control of eating behavior, these BCTs may have contributed to the observed increase in dietary restraint.

The BCTs that may have contributed to the increase in habit strength are social support, restricting the food environment and general information on behavior-health link. These have all been linked to behavioral cueing, a construct that promotes the formation of habits [52, 53]. However, the finding that habit strength was a significant independent mediator for the 52 week intervention but not the 12 week intervention indicates that the intervention length might be an influential moderating factor. This may be linked to a higher “dose” of the BCTs in the 52 week intervention compared to the 12 week intervention due to the longer duration, which may help the formation of stronger habits to support weight maintenance. This formation of stronger habits may be particularly important as piecewise analysis indicated that a reduction in habit strength following the intervention was associated with an increase in BMI. Given that the content of the weight-management programs were the same other than their length, the 52 week intervention provided participants with continued social support from the group leader and other attendees, as well as more opportunity to perform behaviors frequently in a stable context compared to the 12 week intervention; this may have enabled the transition of diet monitoring behavior from deliberative to automatic control [54], which, in turn, supported weight loss maintenance. Such an interpretation is in line with dual-process theories. These theories outline deliberative (or reflective) processes that involve conscious and rational decision-making and automatic (or impulsive) processes that involve nonconscious, learned reactions [55–57]. This is particularly important in health behaviors when individuals aiming to perform healthy behaviors often have to overcome unhealthy habitual behaviors and make conscious and reasoned healthier decisions [56]. These findings support the use of long-term interventions that may facilitate the transition from deliberative attempts to control eating (dietary restraint) to more automatic and less effortful self-regulation of eating behavior (habit strength).

Although autonomous self-regulation was identified as an independent significant mediator for the 52 week intervention, all groups actually experienced a decrease in autonomous motivation throughout the trial and follow-up. This indicates that, although the lesser reduction experienced by the individuals in the 52 week intervention compared to the other two groups was beneficial (for weight loss), all interventions (including the brief intervention) had a negative effect on autonomous self-regulation. It is possible that this, and other, weight-management interventions may have a negative impact on autonomous self-regulation through implicitly promoting the message that participants need to be told what to do by people with expertise in order to manage their weight [28]. This is supported by qualitative findings from the WRAP trial that suggested that participants felt a sense of obligation to the leader of the group sessions [58]. The weight loss and weight loss maintenance achieved in both the 12 and 52 week intervention may have been greater if autonomous self-regulation had been maintained or increased during the intervention.

The findings have implications for the content of future interventions. Given that dietary restraint, habit strength, and autonomous diet self-regulation mediated the effect of the weight-management program on weight loss and maintenance over 2 years, researchers should consider including BCTs that are hypothesized to target these mechanisms of action in future interventions. Recent research that has sought to link specific BCTs and mechanisms of action could be used to identify further BCTs to increase dietary restraint, habit strength, and autonomous diet self-regulation [52, 53]. For example, expert consensus exercises have indicated that the BCTs of introducing prompts and cues for a desired behavior and avoiding or reducing exposure to cues for an unhealthy behavior may be linked to behavioral cueing [52], a mechanism of action that is likely to support the formation of new habits. Similarly, self-monitoring and goal setting have been linked to behavioral regulation [53] and could be used as strategies to support dietary restraint. Although a range of BCTs have been linked with motivation as a mechanism of action, including the use of rewards and the consideration of pros and cons [52, 53], particular attention needs to be given to how to specifically target autonomous motivation. For example, interventions implementing an autonomy-supportive environment, in which individuals are encouraged to engage in health-related behaviors for their own reasons, are supported in overcoming barriers to change, and are made to feel accepted and respected, have been found to be associated with higher autonomous self-regulation, a healthier diet, and greater weight loss in a meta-analysis [59]. In contrast, techniques such as the use of rewards may foster more extrinsic or controlled forms of motivation, which, although may promote initial behavior change, may not be sufficient to support the maintenance of behavior change [60, 61]. In addition, given that the longer duration of intervention was associated with larger changes in dietary restraint and habit strength, researchers should consider interventions that provide support over an extended period of time to promote sustained changes in those mechanisms of action that contribute to weight loss maintenance.

A key strength of this study compared to previous studies was the use of LGCA to disentangle the complex system of interactions between behavioral weight-management interventions, mechanisms of action, and the trajectory of weight change. This method enabled a mediation analysis that accounted for changes at every time point rather than just two time points that are often considered in traditional regression methods. This is particularly important as changes in the mediators and BMI were nonlinear and an analysis assuming a linear trajectory may not have captured the full impact of the mediating variables. This method also enabled growth factors to be both outcomes and predictors. For example, the model tested enabled the slope of the habit strength to be an outcome conditional on treatment group, age, and gender and a predictor of the BMI trajectory simultaneously. These results largely support previous research, which indicates that dietary restraint, habit strength, and self-regulation are potential mediators for the effect of a behavior weight-management program on weight loss and weight loss maintenance [8, 15, 17, 23, 31, 32]. In particular, the findings add to the small number of formal mediation analyses on these factors [17, 27, 33] and, using a complex method examining the mediating action of the three variables simultaneously, provide evidence that these are relevant mechanisms of action for weight management.

There were some study limitations that needed to be taken into consideration when interpreting the findings. First, it was not possible to include the associations between the quadratic growth factors of the mediators and the trajectory of BMI due to nonconvergence of the individual latent growth curves (conducted in Step 1 of the analysis) when allowing the variance of the quadratic factors to vary between individuals. Thus, the rate of acceleration/deceleration of change in BMI was not conditional on the acceleration/deceleration of change (quadratic) of the mediating variables. Including this would have resulted in a greater understanding of the associations between the mediators and BMI. However, even without this, the model fit was adequate. Second, the attrition rate was over 30% at 12 and 24 months, which could have introduced some bias; however, multiple imputation was used, which is a valid general method for managing missing data in RCTs [62]. Finally, although participants were referred to the commercial weight loss program and the cost of sessions was covered for a set period of time (either 12 or 52 weeks), attendance at weekly sessions was not recorded consistently throughout the trial. Due to the large proportion of missing data on attendance (40%), it was not included as a covariate in the analysis. Therefore, the potential impact of attendance on both the mediators and BMI was not controlled for.

In conclusion, dietary restraint, habit strength, and autonomous diet self-regulation were all identified as mechanisms of action for the effective 52 week weight-management program. The finding that habit strength was only a significant mediator of the 52 week program suggests that longer interventions may provide the consistency of support required for behaviors to move from deliberative to habitual control. BCTs that target dietary restraint and habit strength and maintain or increase autonomous diet self-regulation should be considered when designing weight loss and weight loss maintenance interventions.

## Supplementary Material

kaab019_suppl_Supplementary_Material
