# Supplementary material for: Mechanisms of Action in a Behavioral Weight-Management Program: Latent Growth Curve Analysis
Source: Ann Behav Med. 2021 Apr 3;56(1):64–77. doi: 10.1093/abm/kaab019 (PMC8691398; doi:10.1093/abm/kaab019)
Supplement: kaab019_suppl_Supplementary_Material [file kaab019_suppl_supplementary_material.docx]

Supplementary Material

[Participants 2](#_Toc62729385)

[Descriptive Analysis 3](#_Toc62729386)

[Missing Data 4](#_Toc62729387)

[Latent growth curve analysis 7](#_Toc62729388)

[Results 13](#_Toc62729389)

[Table 1. Mean values of psychological variables in each treatment group at each time points 2](#_Toc26182261)

[Table 2. Percentage of participants that completed each assessment after baseline 3](#_Toc26182262)

[Table 3. The percentage of missing data at each time point of individual measures 3](#_Toc26182263)

[Table 4. The percentage of missing data across all groups 3](#_Toc26182264)

[Table 5. Latent growth curve analysis of BMI 10](#_Toc26182265)

[Table 6. Latent growth curve analysis of habit 10](#_Toc26182266)

[Table 7. Latent growth curve analysis of dietary restraint 11](#_Toc26182267)

[Table 8. Latent growth curve analysis of autonomous diet self-regulation 11](#_Toc26182268)

[Table 9. Latent growth curve analysis of controlled diet self-regulation 12](#_Toc26182269)

[Table 10. Latent growth curve analysis of amotivation diet self-regulation 12](#_Toc26182270)

[Table 11. Standardised total, direct and indirect effects via mediating variables of the 12- and 52-week intervention on BMI 14](#_Toc26182271)

[Figure 2. Prediction matric used in multiple imputation 4](#_Toc26182330)

[Figure 3. Unconditional latent growth curve model 6](#_Toc26182331)

[Figure 4. Mediator conditional growth curve model 7](#_Toc26182332)

[Figure 5. BMI conditional growth curve model 7](#_Toc26182333)

[Figure 6. Full mediation model tested 13](#_Toc26182334)

Participants


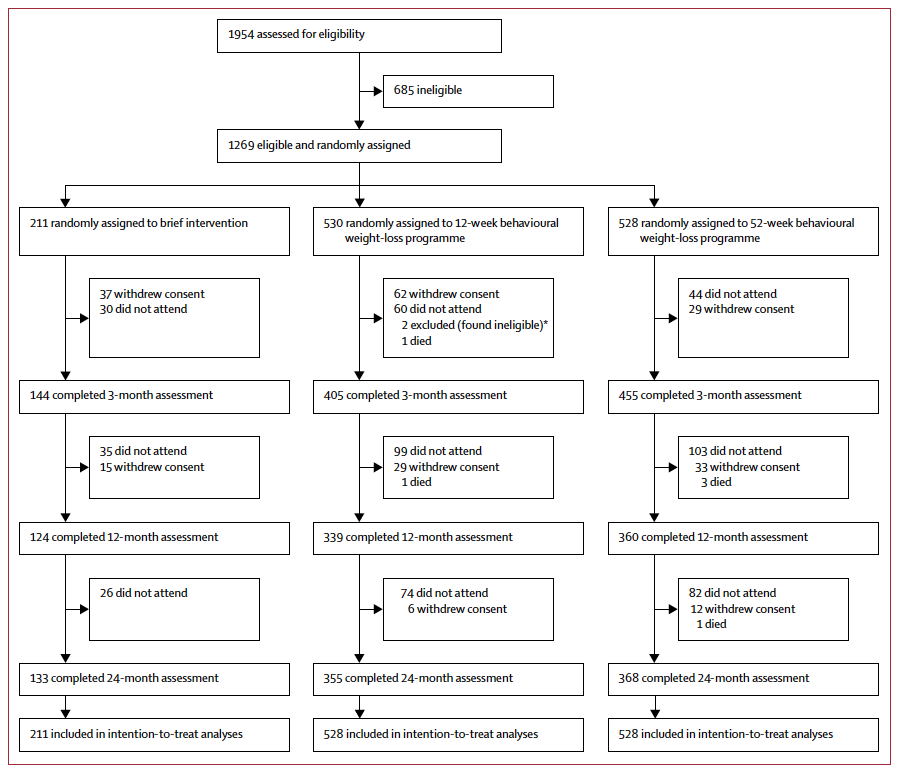


*Excluded from intention-to-treat analyses.

Figure 1: Trial profile [Ahern AL, Wheeler GM, Aveyard P, et al. Extended and standard duration weight-loss programme referrals for adults in primary care (WRAP): a randomised controlled trial. The Lancet. 2017; 389:2214-2225.]

Descriptive Analysis

Table 1. Mean values of psychological variables in each treatment group at each time points

| Time | Treatment group | Dietary restraint | | Diet self-regulation | | | | | | Habit | | |
| --- | --- | --- | --- | --- | --- | --- | --- | --- | --- | --- | --- | --- |
|  |  | Mean | sd | Autonomous | | Controlled | | Motivation | | Mean | sd |  |
|  |  |  |  | Mean | sd | Mean | sd | Mean | sd |  |  |  |
| Baseline | BI | 5.39 | 3.26 | 5.93 | 1.07 | 3.55 | 1.47 | 2.41 | 1.14 | 3.24 | 1.38 |  |
|  | 12 weeks | 4.88 | 3.03 | 5.99 | 0.92 | 3.32 | 1.39 | 2.39 | 1.10 | 3.08 | 1.29 |  |
|  | 52 weeks | 5.34 | 3.06 | 6.04 | 0.97 | 3.44 | 1.36 | 2.40 | 1.09 | 3.14 | 1.38 |  |
| 3 months | BI | 7.55 | 3.13 | 5.71 | 1.15 | 3.44 | 1.49 | 2.52 | 1.23 | 3.64 | 1.33 |  |
|  | 12 weeks | 8.20 | 3.10 | 5.82 | 1.04 | 3.24 | 1.34 | 2.29 | 1.05 | 3.72 | 1.32 |  |
|  | 52 weeks | 8.66 | 2.89 | 5.95 | 1.07 | 3.30 | 1.33 | 2.31 | 1.09 | 3.84 | 1.31 |  |
| 12 months | BI | 6.84 | 3.28 | 5.43 | 1.32 | 3.21 | 1.43 | 2.63 | 1.31 | 3.51 | 1.28 |  |
|  | 12 weeks | 7.23 | 3.22 | 5.60 | 1.17 | 3.08 | 1.29 | 2.34 | 1.06 | 3.66 | 1.33 |  |
|  | 52 weeks | 8.39 | 3.14 | 5.73 | 1.17 | 3.27 | 1.34 | 2.35 | 1.10 | 3.87 | 1.32 |  |
| 24 months | BI | 6.50 | 3.00 | 5.23 | 1.32 | 3.30 | 1.43 | 2.49 | 1.10 | 3.67 | 1.42 |  |
|  | 12 weeks | 6.46 | 3.35 | 5.48 | 1.23 | 3.04 | 1.28 | 2.32 | 1.04 | 3.55 | 1.39 |  |
|  | 52 weeks | 7.25 | 3.39 | 5.45 | 1.31 | 3.16 | 1.36 | 2.38 | 1.10 | 3.71 | 1.33 |  |

Missing Data

**Method**

Forty imputations were generated based on the rule of thumb that number of imputations should match the average percentage rate of missingness (Bodner, 2008). In this case the amount of missing data was highest at year 3 (35%) and so the number of imputations was rounded up to 40 to ensure that there was a sufficient amount. The method chosen for the continuous variable was predictive mean matching which is a semi-parametric method which restricts the imputed values to the observed values and preserves non-linear relationships between the variables used to impute the missing data. Multinomial logit model were used for the categorial variables (Buuren & Groothuis-Oudshoorn, 2010). As recommended (Bodner, 2008), 30 iterations were conducted for each imputation.

**Variables used for prediction**

It was assumed that gender and age predicted the missing data of the potential mediator variables as it is recommended that covariates used in analysis are also used in prediction of missing data. These, income and education predicted the missing data of the BMI variable based on evidence that these factors impact on BMI (Tyrrell et al., 2016). Treatment group was also used to impute missing values of BMI and psychological variables at 3, 12 and 24 months and missing values of variables and each time point were predicted by values of that variables at other time points. Any variables that were correlated (with a correlation of at least .30) and had enough usable cases to predict missing values in the other variable were also retained as predictors.

Table 2. Percentage of participants that completed each assessment after baseline

| Time after baseline | Brief intervention | 12-week intervention | 52-week intervention | All groups |
| --- | --- | --- | --- | --- |
| 3 months | 68 | 77 | 86 | 79 |
| 12 months | 59 | 64 | 68 | 65 |
| 24 months | 63 | 67 | 70 | 68 |

Table 3. The percentage of missing data at each time point for each measure

| Time | Treatment group | BMI | Dietary restraint | Diet self-regulation | | | Habit |
| --- | --- | --- | --- | --- | --- | --- | --- |
|  |  |  |  | Autonomous | Controlled | Amotivation |  |
| Baseline | BI | 0.00 | 3.32 | 3.79 | 3.32 | 3.32 | 3.32 |
|  | 12 weeks | 0.00 | 2.27 | 2.65 | 2.27 | 2.46 | 2.65 |
|  | 52 weeks | 0.00 | 2.27 | 2.27 | 2.46 | 2.65 | 3.03 |
| 3 months | BI | 31.75 | 37.91 | 39.34 | 38.39 | 38.86 | 38.86 |
|  | 12 weeks | 23.30 | 28.03 | 28.79 | 28.98 | 28.98 | 29.17 |
|  | 52 weeks | 13.83 | 17.61 | 19.51 | 19.32 | 19.32 | 19.32 |
| 12 months | BI | 41.23 | 49.76 | 49.29 | 49.76 | 50.24 | 49.76 |
|  | 12 weeks | 35.80 | 39.39 | 39.39 | 39.77 | 39.96 | 39.96 |
|  | 52 weeks | 31.82 | 36.17 | 36.55 | 36.74 | 36.74 | 36.93 |
| 24 months | BI | 36.97 | 45.50 | 45.50 | 46.92 | 47.39 | 46.92 |
|  | 12 weeks | 32.77 | 39.58 | 39.77 | 41.10 | 41.10 | 41.48 |
|  | 52 weeks | 30.30 | 38.07 | 38.45 | 38.07 | 38.07 | 38.26 |

Table 4. The percentage of missing data across all groups

| Time | BMI | Dietary restraint | Diet self-regulation | | | Habit |
| --- | --- | --- | --- | --- | --- | --- |
|  |  |  | Autonomous | Controlled | Motivation |  |
| Baseline | 0.00 | 2.62 | 2.91 | 2.68 | 2.81 | 3.00 |
| 12 weeks | 22.96 | 27.85 | 29.21 | 28.89 | 29.05 | 29.12 |
| 52 weeks | 36.28 | 41.78 | 41.75 | 42.09 | 42.31 | 42.22 |
| 3 months | 33.35 | 41.05 | 41.24 | 42.03 | 42.19 | 42.22 |

Figure 2. Prediction matric used in multiple imputation

|  | SerNo | TXGROUP | Sex | INCOME | EDU | AGE | BMI.0 | DRES.0 | HABIT.0 | DSRA.0 | DSRC.0 | DSRM.0 | BMI.3 | DRES.3 | HABIT.3 | DSRA.3 | DSRC.3 | DSRM.3 | BMI.12 | DRES.12 | HABIT.12 | DSRA.12 | DSRC.12 | DSRM.12 | BMI.24 | DRES.24 | HABIT.24 | DSRA.24 | DSRC.24 | DSRM.24 |
| --- | --- | --- | --- | --- | --- | --- | --- | --- | --- | --- | --- | --- | --- | --- | --- | --- | --- | --- | --- | --- | --- | --- | --- | --- | --- | --- | --- | --- | --- | --- |
| TXGROUP | 0 | 0 | 0 | 0 | 0 | 0 | 0 | 0 | 0 | 0 | 0 | 0 | 0 | 0 | 0 | 0 | 0 | 0 | 0 | 0 | 0 | 0 | 0 | 0 | 0 | 0 | 0 | 0 | 0 | 0 |
| Sex | 0 | 0 | 0 | 0 | 0 | 0 | 0 | 0 | 0 | 0 | 0 | 0 | 0 | 0 | 0 | 0 | 0 | 0 | 0 | 0 | 0 | 0 | 0 | 0 | 0 | 0 | 0 | 0 | 0 | 0 |
| INCOME | 0 | 1 | 1 | 0 | 1 | 1 | 0 | 0 | 0 | 0 | 0 | 0 | 0 | 0 | 0 | 0 | 0 | 0 | 0 | 0 | 0 | 0 | 0 | 0 | 0 | 0 | 0 | 0 | 0 | 0 |
| EDU | 0 | 1 | 1 | 1 | 0 | 1 | 0 | 0 | 0 | 0 | 0 | 0 | 0 | 0 | 0 | 0 | 0 | 0 | 0 | 0 | 0 | 0 | 0 | 0 | 0 | 0 | 0 | 0 | 0 | 0 |
| AGE | 0 | 1 | 1 | 1 | 0 | 1 | 0 | 0 | 0 | 0 | 0 | 0 | 0 | 0 | 0 | 0 | 0 | 0 | 0 | 0 | 0 | 0 | 0 | 0 | 0 | 0 | 0 | 0 | 0 | 0 |
| BMI.0 | 0 | 0 | 1 | 1 | 1 | 1 | 0 | 0 | 0 | 0 | 0 | 0 | 1 | 0 | 0 | 0 | 0 | 0 | 1 | 0 | 0 | 0 | 0 | 0 | 1 | 0 | 0 | 0 | 0 | 0 |
| DRES.0 | 0 | 0 | 1 | 1 | 0 | 1 | 0 | 0 | 0 | 0 | 0 | 0 | 0 | 1 | 1 | 0 | 0 | 0 | 0 | 1 | 0 | 0 | 0 | 0 | 0 | 1 | 0 | 0 | 0 | 0 |
| HABIT.0 | 0 | 0 | 1 | 1 | 0 | 1 | 0 | 0 | 0 | 0 | 0 | 0 | 0 | 0 | 1 | 0 | 0 | 0 | 0 | 0 | 1 | 0 | 0 | 0 | 0 | 0 | 1 | 0 | 0 | 0 |
| DSRA.0 | 0 | 0 | 1 | 1 | 0 | 1 | 0 | 0 | 0 | 0 | 0 | 0 | 0 | 0 | 0 | 1 | 0 | 0 | 0 | 0 | 0 | 1 | 0 | 0 | 0 | 0 | 0 | 1 | 0 | 0 |
| DSRC.0 | 0 | 0 | 1 | 1 | 0 | 1 | 0 | 0 | 0 | 0 | 0 | 0 | 0 | 0 | 0 | 0 | 1 | 1 | 0 | 0 | 0 | 0 | 1 | 1 | 0 | 0 | 0 | 0 | 1 | 0 |
| DSRM.0 | 0 | 0 | 1 | 1 | 0 | 1 | 0 | 0 | 0 | 0 | 0 | 0 | 0 | 0 | 0 | 0 | 1 | 1 | 0 | 0 | 0 | 0 | 1 | 1 | 0 | 0 | 0 | 0 | 1 | 1 |
| BMI.3 | 0 | 1 | 1 | 1 | 0 | 1 | 1 | 0 | 0 | 0 | 0 | 0 | 0 | 0 | 0 | 0 | 0 | 0 | 1 | 0 | 0 | 0 | 0 | 0 | 1 | 0 | 0 | 0 | 0 | 0 |
| DRES.3 | 0 | 1 | 1 | 1 | 0 | 1 | 0 | 1 | 0 | 0 | 0 | 0 | 0 | 0 | 0 | 0 | 0 | 0 | 0 | 1 | 0 | 0 | 0 | 0 | 0 | 1 | 0 | 0 | 0 | 0 |
| HABIT.3 | 0 | 1 | 1 | 1 | 0 | 1 | 0 | 1 | 1 | 0 | 0 | 0 | 0 | 0 | 0 | 0 | 0 | 0 | 0 | 0 | 1 | 0 | 0 | 0 | 0 | 0 | 1 | 0 | 0 | 0 |
| DSRA.3 | 0 | 1 | 1 | 1 | 0 | 1 | 0 | 0 | 0 | 1 | 0 | 0 | 0 | 0 | 0 | 0 | 0 | 0 | 0 | 0 | 0 | 1 | 0 | 0 | 0 | 0 | 0 | 1 | 0 | 0 |
| DSRC.3 | 0 | 1 | 1 | 1 | 0 | 1 | 0 | 0 | 0 | 0 | 1 | 1 | 0 | 0 | 0 | 0 | 0 | 0 | 0 | 0 | 0 | 0 | 1 | 0 | 0 | 0 | 0 | 0 | 1 | 0 |
| DSRM.3 | 0 | 1 | 1 | 1 | 0 | 1 | 0 | 0 | 0 | 0 | 1 | 1 | 0 | 0 | 0 | 0 | 0 | 0 | 0 | 0 | 0 | 0 | 0 | 1 | 0 | 0 | 0 | 0 | 0 | 1 |
| BMI.12 | 0 | 1 | 1 | 1 | 0 | 1 | 1 | 0 | 0 | 0 | 0 | 0 | 1 | 0 | 0 | 0 | 0 | 0 | 0 | 0 | 0 | 0 | 0 | 0 | 1 | 0 | 0 | 0 | 0 | 0 |
| DRES.12 | 0 | 1 | 1 | 1 | 0 | 1 | 0 | 1 | 0 | 0 | 0 | 0 | 0 | 1 | 1 | 1 | 0 | 0 | 0 | 0 | 0 | 0 | 0 | 0 | 0 | 1 | 0 | 0 | 0 | 0 |
| HABIT.12 | 0 | 1 | 1 | 1 | 0 | 1 | 0 | 0 | 1 | 0 | 0 | 0 | 0 | 0 | 1 | 0 | 0 | 0 | 0 | 0 | 0 | 0 | 0 | 0 | 0 | 0 | 1 | 0 | 0 | 0 |
| DSRA.12 | 0 | 1 | 1 | 1 | 0 | 1 | 0 | 0 | 0 | 1 | 0 | 0 | 0 | 0 | 0 | 1 | 0 | 0 | 0 | 0 | 0 | 0 | 0 | 0 | 0 | 0 | 0 | 1 | 0 | 0 |
| DSRC.12 | 0 | 1 | 1 | 1 | 0 | 1 | 0 | 0 | 0 | 0 | 1 | 1 | 0 | 0 | 0 | 0 | 1 | 1 | 0 | 0 | 0 | 0 | 0 | 0 | 0 | 0 | 0 | 0 | 1 | 0 |
| DSRM.12 | 0 | 1 | 1 | 1 | 0 | 1 | 0 | 0 | 0 | 0 | 1 | 1 | 0 | 0 | 0 | 0 | 1 | 1 | 0 | 0 | 0 | 0 | 0 | 0 | 0 | 0 | 0 | 0 | 0 | 1 |
| BMI.24 | 0 | 1 | 1 | 1 | 0 | 1 | 1 | 0 | 0 | 0 | 0 | 0 | 1 | 0 | 0 | 0 | 0 | 0 | 1 | 0 | 0 | 0 | 0 | 0 | 0 | 0 | 0 | 0 | 0 | 0 |
| DRES.24 | 0 | 1 | 1 | 1 | 0 | 1 | 0 | 1 | 0 | 0 | 0 | 0 | 0 | 1 | 0 | 0 | 0 | 0 | 0 | 1 | 0 | 0 | 0 | 0 | 0 | 0 | 0 | 0 | 0 | 0 |
| HABIT.24 | 0 | 1 | 1 | 1 | 0 | 1 | 0 | 0 | 1 | 0 | 0 | 0 | 0 | 0 | 1 | 0 | 0 | 0 | 0 | 0 | 1 | 0 | 0 | 0 | 0 | 0 | 0 | 0 | 0 | 0 |
| DSRA.24 | 0 | 1 | 1 | 1 | 0 | 1 | 0 | 0 | 0 | 1 | 0 | 0 | 0 | 0 | 0 | 1 | 0 | 0 | 0 | 0 | 0 | 1 | 0 | 0 | 0 | 0 | 0 | 0 | 0 | 0 |
| DSRC.24 | 0 | 1 | 1 | 1 | 0 | 1 | 0 | 0 | 0 | 0 | 1 | 1 | 0 | 0 | 0 | 0 | 1 | 1 | 0 | 0 | 0 | 0 | 1 | 0 | 0 | 0 | 0 | 0 | 0 | 0 |
| DSRM.24 | 0 | 1 | 1 | 1 | 0 | 1 | 0 | 0 | 0 | 0 | 0 | 1 | 0 | 0 | 0 | 0 | 0 | 1 | 0 | 0 | 0 | 0 | 0 | 1 | 0 | 0 | 0 | 0 | 0 | 0 |

Latent growth curve analysis

In latent growth curve analysis (LGCA), the value for each individual (i) at each time point (t) can be represented by a linear (Eq. 1) or quadratic (Eq. 2) equations where $\mathbb{I, S}$ and $\mathbb{Q}$ are the intercept, slope and quadratic latent growth factors respectively assuming that the error term is normally and independently distributed.

|  | $Variable_{it}= \mathbb{I}_{i}+ \mathbb{S}_{i} t+ \varepsilon_{it}$ | 1 |
| --- | --- | --- |
|  | $Variable_{it}= \mathbb{I}_{i}+ \mathbb{S}_{i} t\mathbb{+Q}t^{2}+ \varepsilon_{it}$ | 2 |

The growth factors of each individual is used to estimate the average growth factors and an aggregated error variance for each as well as correlation between the growth factors(Wickrama et al., 2016).

*Analysis Strategy*

First, we fitted an unconditional model. We tested increasingly complex models starting with a model in which its assumed that all participants have the same intercept and then testing each of the hypothesis below following recommendations in the literature (Preacher et al., 2008).

1. There will be variation between individual in the level of the variable
2. There will be a change in the variables over the course of two years
3. There will be variation in individuals in the extent of change in the variable over the two years
4. There will be a non-linear (quadratic) change in the variable over the two years
5. There will be a variation in individuals in the extent of non-linear (quadratic) change in the variable over the 2 years.

The factor loadings (represented as coefficients) for the intercept were set to 1. The coefficient of the slopes reflect the time points at which the data was collected in months (0, 3, 12, 24) and similarly the quadratic represent the acceleration or deceleration of changes and the loading and the squared values of those used for the slope (0, 9, 144, 576) (Wickrama et al., 2016).

For each of them the nested $\mathcal{X}$ ^2^ difference test ($\mathcal{X}$^2^_DIFF_) was used to compare models (the more complex model to the previous one tested). A significant value on this test indicates that the more complex model is a better fit than he less complex model. If the value is not significant then both models fit equally well and thus the simple model is considered the preferred option (Wickrama et al., 2016). However, all model fit indices will be considered when deciding on the best fitting model. An example of a growth model with the maximum amount of growth factors possible for this data set (intercept, slope and quadratic) is shown in figure 3.


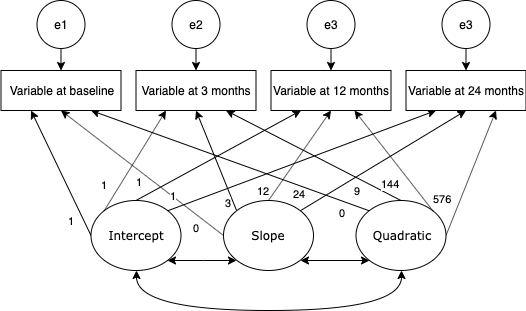


Figure 3. Unconditional latent growth curve model

Once model fit was established for the unconditional model, a conditional model was fitted (Preacher et al., 2008). These included age and gender for all variables and education and income for BMI. Treatment group was included as a covariate for the slope and quadratic (due to randomisation, no impact of group allocation on intercept was modelled). All variables were treated as time-invariant; although age is a time-variant variable in reality, as the trial was only two years long, we used starting age as a time-invariant variable to avoid additional complexity in the model. For the psychological variables, the estimates of each of the growth factors in show in equations 3-5 where $\alpha$, $\beta$ and $\gamma$ are the coefficients linking the age, sex and treatment group to the growth factor. The same for the BMI variables in in equations 6-8 where $\rho$ and $\nu$ are the coefficients linking income and education to the latent growth factors. Conditional growth models for potential mediators and BMI are shown in Figure 4 and 5 respectively.

|  | $\mathbb{I}_{i}=\mu_{00}+ \alpha_{0} Age_{i}+ \beta_{0} Sex_{i}$ | 3 |
| --- | --- | --- |
|  | $\mathbb{S}_{i}=\mu_{00}+ \alpha_{1} Age_{i}+ \beta_{1} Sex_{i}+ \gamma_{1} TG$ | 4 |
|  | $\mathbb{Q}_{i}=\mu_{00}+ \alpha_{2} Age_{i}+ \beta_{2} Sex_{i}+ \gamma_{2} TG$ | 5 |
|  | $\mathbb{I}_{i}=\mu_{00}+ \alpha_{0} Age_{i}+ \beta_{0} Sex_{i}+ \rho_{0}Income_{i}+ \nu_{0}Education_{i}$ | 6 |
|  | $\mathbb{S}_{i}=\mu_{00}+ \alpha_{1} Age_{i}+ \beta_{1} Sex_{i}+ \rho_{0}Income_{i}+ \nu_{0}Education_{i}+ \gamma_{1} TG$ | 7 |
|  | $\mathbb{Q}_{i}=\mu_{00}+ \alpha_{2} Age_{i}+ \beta_{2} Sex_{i} + \rho_{0}Income_{i}+ \nu_{0}Education_{i}+ \gamma_{2} TG$ | 8 |


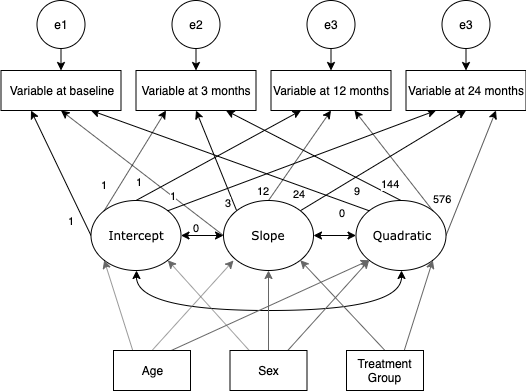


Figure 4. Mediator conditional growth curve model


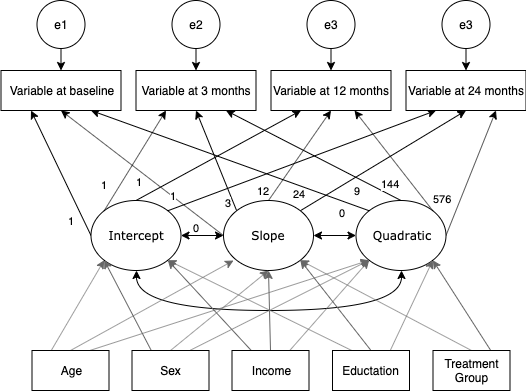


Figure 5. BMI conditional growth curve model

*Piecewise growth curve model*

Piecewise growth curve model were also fitted to the trajectories of each of the mechanisms of action and BMI as a secondary analysis. This enables a single intercept with two slopes which can be used to represent the intervention stage (up to 12 months) and the maintenance stage (12-24 months). Ideally piecewise analysis requires at least 5 time points such that three time points can be used for each curve (Wickrama et al., 2016). However piecewise analysis can be conducted on fewer time points, although the requires restriction on some parameters (for example, limiting the variance of, or covariance between, growth factors to zero) to allow the model to be fitted (Kamata et al., 2013). These were fitted at each stage following the same procedure as described in the analysis strategy however based on the findings from the latent growth curve analysis, it was assumed that there were two slope factors. The piecewise model was fitted with each of 3 and 12 months as the points that the slopes meet to test which was the better fit (steps 1 and 2). The slope of the first slope was varied first as the change in the variable was the focus and greater variation change was expected in this phase when the intervention took place. The model with the better fit was taken through to the next stage:

1. Intercept and both slopes fixed to zero, slope 1 is 0-3 months, slope 2 is 3-24 months
2. Intercept and both slopes fixed to zero, slope 1 is 0-12 months, slope 2 is 12-24 months
3. Intercept and slope 2 fixed to zero
4. Intercept fixed to zero

The unconditional and conditional examples of a piecewise model are shown in Figures 6 and 7.


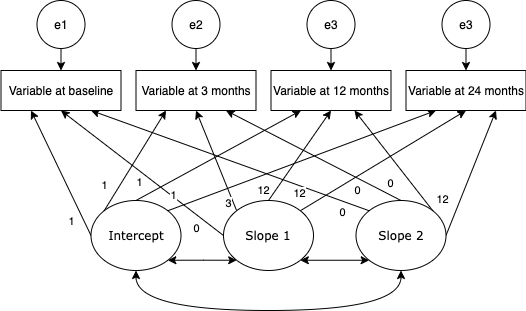


Figure 6. Mediator unconditional piecewise growth curve model


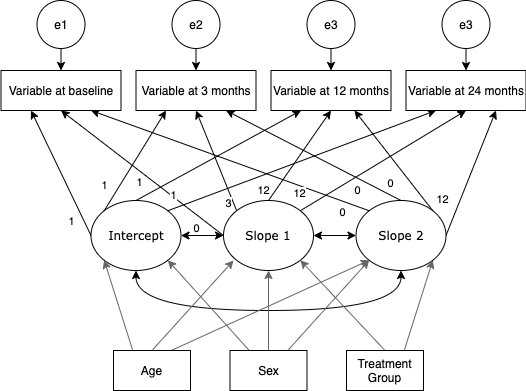


Figure 7. Mediator conditional piecewise growth curve model

*Benefits of the method*

LGCA as a method has many benefits over traditional regression methods, an alternative analysis method for this type of research question. Standard regression methods tend to use just two time points, the baseline and the last time point. The last time point is regressed on the predictors while controlling for the baseline value; thus, the analysis examines predictors of residual values (final value with the effect of the baseline value removed) (Wickrama et al., 2016). When there are more time points available, as in the data set from the WRAP study, the information available about values about time points between the first and last is lost. In addition, if the trajectory of variables is not linear, the impact of covariate may not be detected (Wickrama et al., 2016). This is important for this analysis as the intervention takes place within the first year and therefore it is reasonable to expect an initial change from baseline and a return towards the baseline value. This does not reflect a linear trajectory.

Another benefit of LGCA is that it is possible to investigate the different growth parameters in one analysis (Preacher et al., 2008). Although in this analysis, we were most interested in the change over time (slope and quadratic factors), we were also able to account for any association between the change in the outcome variable (BMI) and baseline values of the predictors (intercept) as well as the impact of the change in predictors on the change in BMI. This allows a more complete picture of the association between the predictors and the outcome. In addition, the growth factors can be used as predictor or outcomes. In the mediation model, slopes of the psychological variables were an outcome (predicted by demographic variables and group allocation) and predictors (of change in BMI).

Finally, the ability of enabling a time-varying variable (in this case the slope of the mediator) to be a predictor of another time-varying variable (slope and quadratic of BMI) in both the parallel processing model (step 2) and the full mediation would be challenging to do with other regression models.

*Limitations*

There are some limitations of this method. The shape of the curve is expected to be the same for all individuals and so although the covariate can reflect the magnitude of the growth factors, they cannot be used to predict shape. This is a limitation in this study as the three groups had different intervention time periods and therefore different curve shapes was possible (Wickrama et al., 2016). However, as can be seen in figure 1 in the main paper, the trajectory of change in the groups seem to follow a similar shape but with different magnitudes and thus we don’t expect this limitation to have had a large impact. The analysis is based on the assumption that variables are univariate and multivariate normally distributed (Preacher et al., 2008). This was initially a concern because the variables used were not normally distributed. However, we used a method of estimation method of that is robust to non-normal distributions and found that the results did not differ significantly from the standard methods. Finally, it’s acknowledged that the fit of a model isn’t easy to asses as there is not a single measure of fit and researchers must make a decision. Therefore, throughout the results, we included model fit statistics and have highlighted when some are below recommended cut off values.

*Considerations*

1. Measurement issues

Often in LGCA, the observation between time points will be correlated such that time points closer together will have a stronger correlation than time points further apart (Singer & Willett, 2003). However, in this study, this may not be the case as we could expect more change between baseline and 3 months in which all participants have an intervention of some kind than between year 1 and 2 when there is no reported intervention. Thus, in this LGCA we’ve not added correlation paths between adjacent and nearly adjacent variables. Another potential measurement issue is the assumption of homoscedasticity over time (Van de Schoot et al., 2012). That is, is it expected that at each time point the residual variance around an observation will be the same (i.e. the is the same around of variation among individuals at each time point). Thus, changes observed between time points can be assumed to be changes in in these variables rather than changes in residual variance. When fitting a curve to each of the variables, we fixed variances for each time to be equal. For each variable we also checked whether the model fit was better without this fixing to determine any problems with this assumption.

1. Sample size

The sample size in terms of the number of time points and number of participants impacts has an impact on LGCA. The number of time points determine the complexity of the growth curve through the number of growth factors. The number of growth factors much be at least one less than the number of time points. In the case, because there were four time points, the maximum number of growth factors was three (intercept, slope and quadratic). There are no clear guidelines on the number of time points needed (MacCallum et al., 1997). Some suggest that four to five measurements are sufficient and but that this is conditional on effect size, sample size and sample size (Hertzog et al., 2006), others recommends that a limited number occasions avoid high levels of complexity that can make achieving an adequate fit challenging (Preacher et al., 2008). The important point is that the time points should adequate cover the time of interest. In this case, the time points were before intervention, after one group finished their intervention, after the second group finished the intervention and then one year later which covers the points in time where the most change was expected to occur.

In terms of the number of subjects, again there is not clear guidelines for this although at least 100 are preferred (Curran et al., 2010)^,^(Hamilton et al., 2003) and our sample exceeded this substantially. However, as the sample size increases, there is a greater probability of rejecting the models based on the significance of the Chi square statistic and therefore this model fit statistic was not used.

Results

In tables 5-10 below the first model tested (0) will be the base model in which it’s assumed that all participants have the same intercept and no change over time. Models 1-5 represent increasingly complex models tested (outlined in previous section). A conditional model (adapted from the best fitting unconditional model) was then fitted. Finally, we checked the homoscedasticity assumption by removing the restriction of equal variances across timepoints.

Tables 11-14 show the model statistics for the piecewise models fitted to BMI and each of the mediators. Models 1-4 are those described previously in the analysis section. A conditional model (adapted from the best fitting unconditional model) was then fitted. Unlike the previous analyses, the homoscedasticity was assumed throughout the models. This is based on the finding that assuming the same variance across time points did not negatively impact model fit in the previous section and because the piecewise model required more parameters to be fixed to avoid oversaturation on the model.

Table 5. Latent growth curve analysis of BMI

| Model | Fit statistics | | | | | Intercept | | Slope | | Quadratic | | Covariance | | |
| --- | --- | --- | --- | --- | --- | --- | --- | --- | --- | --- | --- | --- | --- | --- |
|  | Chi-Square (df) | Change in Chi-Square (df) | RMSEA | CFI | SRMR | mean | variance | mean | variance | mean | variance | Intercept, slope | Intercept, quadratic | Slope, quadratic |
| 0 | 3903.24 (12) |  | 0.51 | 0.00 | 0.62 | 33.38 |  |  |  |  |  |  |  |  |
| 1 | 1005.78 (11) | 2897.46 (1)*** | 0.27 | 0.62 | 0.20 | 33.38 | 24.34 |  |  |  |  |  |  |  |
| 2 | 932.43 (10) | 73.35 (1)*** | 0.27 | 0.64 | 0.17 | 33.69 | 25.27 | -0.32 |  |  |  |  |  |  |
| 3 | 715.38 (8) | 217.05 (2)*** | 0.26 | 0.73 | 0.11 | 33.69 | 24.21 | -0.32 | 0.89 |  |  | 0.24 |  |  |
| 4 | 384.31 (7) | 331.07 (1)*** | 0.21 | 0.85 | 0.06 | 34.22 | 24.56 | -2.69 | 1.08 | 0.98 |  | 0.05 |  |  |
| 5 | 224.35 (4) | 159.96 (3)*** | 0.21 | 0.92 | 0.03 | 34.22 | 25.32 | -2.69 | 10.342 | 0.98 | 1.00 | -2.05* | 0.64* | -3.08* |
| Conditional | 364.35 (15) | NA | 0.14 | 0.93 | 0.02 | 36.16 | 24.50 | 0.84 | 9.52 | 0.03 | 0.92 | -2.15* | 0.67* | -2.82* |
| Check homoscedasticity | 391.21 (12) |  | 0.16 | 0.93 | 0.03 | 36.24 | 24.62 | 0.45 | 7.65 | 0.11 | 1.38 | -2.09 | 0.68 | -2.68 |

Table 6. Latent growth curve analysis of habit strength

| Model | Fit statistics | | | | | Intercept | | Slope | | Quadratic | | Covariance | | | | |
| --- | --- | --- | --- | --- | --- | --- | --- | --- | --- | --- | --- | --- | --- | --- | --- | --- |
|  | Chi-Square (df) | Change in Chi-Square (df) | RMSEA | CFI | SRMR | mean | variance | mean | variance | mean | variance | Intercept, slope | Intercept, quadratic | Slope, quadratic |  |  |
| 0 | 1134.12 (12) |  | 0.27 | 0.00 | 0.39 | 3.84 |  |  |  |  |  |  |  |  |  |  |
| 1 | 340.49 (11) | 793.63 (1)*** | 0.15 | 0.56 | 0.17 | 3.84 | 1.16 |  |  |  |  |  |  |  |  |  |
| 2 | 299.61 (10) | 40.99 (2) *** | 0.15 | 0.62 | 0.17 | 3.70 | 1.17 | 0.14 |  |  |  |  |  |  |  |  |
| 3 | 127.62 (8) | 171.99 (2)*** | 0.12 | 0.84 | 0.11 | 3.40 | 1.22 | 0.28 | 0.01 |  |  | -0.02 |  |  |  |  |
| 4 | 32.95 (7) | 94.67 (2)*** | 0.07 | 0.96 | 0.05 | 3.26 | 1.21 | 0.99 | 0.09 | -0.30 |  | -0.04 |  |  |  |  |
| 5 | Not converged | |  |  |  |  |  |  |  |  |  |  |  |  |  |  |
| Conditional | 41.52 (13) | NA | 0.05 | 0.97 | 0.04 | 1.94 | 1.14 | 0.79 | 0.10 | -0.21 |  | -0.06 |  |  |  |  |
| Check homoscedasticity | 47.77 (10) | NA | 0.05 | 0.98 | 0.03 | 2.28 | 1.09 | 0.63 | -0.06 | -0.17 |  | -0.03 |  |  | |  |

Table 7. Latent growth curve analysis of dietary restraint

| Model | Fit statistics | | | | | Intercept | | Slope | | Quadratic | | Covariance | | |
| --- | --- | --- | --- | --- | --- | --- | --- | --- | --- | --- | --- | --- | --- | --- |
|  | Chi-Square (df) | Change in Chi-Square (df) | RMSEA | CFI | SRMR | mean | variance | mean | variance | mean | variance | Intercept, slope | Intercept, quadratic | Slope, quadratic |
| 0 | 1345.75 (12) |  | 0.30 | 0.00 | 0.40 | 6.96 |  |  |  |  |  |  |  |  |
| 1 | 756.84 (11) | 588.91 (1)*** | 0.23 | 0.05 | 0.25 | 6.96 | 4.50 |  |  |  |  |  |  |  |
| 2 | 311.20 (10) | 445.64 (2)*** | 0.16 | 0.62 | 0.14 | 5.69 | 4.80 | 0.70 |  |  |  |  |  |  |
| 3 | 292.71 (8) | 18.49 (2)*** | 0.18 | 0.64 | 0.14 | 5.69 | 4.31 | 0.71 | -0.09 |  |  | 0.20 |  |  |
| 4 | 34.09 (7) | 258.62 (1)*** | 0.07 | 0.96 | 0.05 | 5.15 | 4.14 | 3.41 | 0.13 | -1.13 |  | 0.43* |  |  |
| 5 | Not converged | |  |  |  |  |  |  |  |  |  |  |  |  |
| Conditional | 45.15 (13) | NA | 0.05 | 0.97 | 0.04 | 2.82 | 3.72 | 2.68 | 0.15 | -0.87 |  | 0.40** |  |  |
| Check homoscedasticity | 38.77 (10) | 6.38 (3)* | 0.04 | 0.98 | 0.03 | 2.82 | 3.53 | 2.68 | 0.37 | -0.86 |  | 0.43** |  |  |

Table 8. Latent growth curve analysis of autonomous diet self-regulation

| Model | Fit statistics | | | | | Intercept | | Slope | | Quadratic | | Covariance | | |
| --- | --- | --- | --- | --- | --- | --- | --- | --- | --- | --- | --- | --- | --- | --- |
|  | Chi-Square (df) | Change in Chi-Square (df) | RMSEA | CFI | SRMR | mean | variance | mean | variance | mean | variance | Intercept, slope | Intercept, quadratic | Slope, quadratic |
| 0 | 381.88 (12) |  | 0.16 | 0.47 | 0.52 | 0.81 |  |  |  |  |  |  |  |  |
| 1 | 359.40 (11) | 22.48 (1)*** | 0.16 | 0.50 | 0.44 | 5.71 | 0.62 |  |  |  |  |  |  |  |
| 2 | 154.57 (10) | 204.83 (1)*** | 0.11 | 0.79 | 0.34 | 5.95 | 0.64 | -0.24 |  |  |  |  |  |  |
| 3 | 55.22 (8) | 99.35 (2)*** | 0.07 | 0.93 | 0.18 | 5.95 | 0.52 | -0.24 | 0.09 |  |  | 0.03 |  |  |
| 4 | 43.72 (7) | 11.50 (1)*** | 0.06 | 0.95 | 0.16 | 5.89 | 0.52 | -0.43 | 0.09 | 0.08 |  | 0.03 |  |  |
| 5 | Not converged | |  |  |  |  |  |  |  |  |  |  |  |  |
| Conditional | 52.49 (13) | NA | 0.05 | 0.96 | 0.02 | 5.75 | 0.51 | -1.16 | 0.09 | 0.36 |  | 0.03 |  |  |
| Check homoscedasticity | 45.57 (10) | 6.92 (3)* | 0.05 | 0.96 | 0.07 | 5.76 | 0.53 | -1.19 | 0.11 | 0.37 |  | 0.01 |  |  |

Table 9. Latent growth curve analysis of controlled diet self-regulation

| Model | Fit statistics | | | | | Intercept | | Slope | | Quadratic | | Covariance | | |
| --- | --- | --- | --- | --- | --- | --- | --- | --- | --- | --- | --- | --- | --- | --- |
|  | Chi-Square (df) | Change in Chi-Square (df) | RMSEA | CFI | SRMR | mean | variance | mean | variance | mean | variance | Intercept, slope | Intercept, quadratic | Slope, quadratic |
| 0 | 1616.59 (12) |  | 0.33 | 0.00 | 0.42 | 3.27 |  |  |  |  |  |  |  |  |
| 1 | 59.23 (11) | 1557.36 (1)*** | 0.06 | 0.96 | 0.03 | 3.27 | 1.18 |  |  |  |  |  |  |  |
| 2 | 25.60 (10) | 33.63 (1)*** | 0.04 | 0.99 | 0.03 | 3.63 | 1.19 | -0.09 |  |  |  |  |  |  |
| 3 | 14.63 (8) | 10.97 (2)** | 0.03 | 1.00 | 0.02 | 3.36 | 1.27 | -0.09 | 0.04 |  |  | -0.06 |  |  |
| 4 | 9.04 (7) | 5.59 (1)* | 0.02 | 1.00 | 0.02 | 3.39 | 1.27 | -0.22 | 0.04 | 0.06 |  | -0.06 |  |  |
| 5* | 5.36 (4) | 3.68 (3) | 0.02 | 1.00 | 0.01 | 3.39 | 1.27 | -0.22 | -0.04 | 0.06 | -0.03 | -0.05 | 0.00 | 0.05 |
| Conditional | 14.76 (13) | NA | 0.02 | 1.00 | 0.02 | 3.10 | 1.26 | -0.08 | -0.09 | 0.09 | -0.03 | -0.04 | -0.1 | 0.07 |
| Check homoscedasticity | 11.08 (10) | 3.68 (2) | 0.02 | 1.00 | 0.02 | 3.08 | 1.22 | -0.06 | -0.17 | 0.09 | -0.05 | 0.04 | -0.03 | 0.10 |

*This model was not significantly better fitting than the previous, more simple, model and therefore the previous model was selected for the next step of the analysis.

Table 10. Latent growth curve analysis of amotivation diet self-regulation

| Model | Fit statistics | | | | | Intercept | | Slope | | | Quadratic | | | Covariance | | |
| --- | --- | --- | --- | --- | --- | --- | --- | --- | --- | --- | --- | --- | --- | --- | --- | --- |
|  | Chi-Square (df) | Change in Chi-Square (df) | RMSEA | CFI | SRMR | mean | variance | mean | variance | mean | | variance | Intercept, slope | | Intercept, quadratic | Slope, quadratic |
| 0 | 802.96 (12) |  | 0.23 | 0.00 | 0.32 | 2.39 |  |  |  |  | |  |  | |  |  |
| 1 | 28.40 (11) | 774.56 (1)*** | 0.04 | 0.97 | 0.04 | 2.39 | 0.59 |  |  |  | |  |  | |  |  |
| 2* | 27.20 (10) | 1.2 (1) | 0.04 | 0.97 | 0.04 | 2.38 | 0.59 | 0.02 |  |  | |  |  | |  |  |
| Conditional | 48.83 (17) | NA | 0.04 | 0.96 | 0.04 | 2.39 | 0.59 |  |  |  | |  |  | |  |  |
| Check homoscedasticity | 42.79 (14) | 6.04 (3) | 0.04 | 0.96 | 0.05 | 2.40 | 0.60 |  |  |  | |  |  | |  |  |

*This model was not significantly better fitting than the previous, more simple, model and therefore the previous model was selected for the next step of the analysis.

Table 11. Piecewise latent growth curve analysis of BMI

| Model | Fit statistics | | | | | Intercept | | Slope | | Slope 2 | | Covariance | | | |
| --- | --- | --- | --- | --- | --- | --- | --- | --- | --- | --- | --- | --- | --- | --- | --- |
|  | Chi-Square (df) | Change in Chi-Square (df) | RMSEA | CFI | SRMR | mean | variance | mean | variance | mean | variance | Intercept, slope | Intercept, slope 2 | Slope 2, slope 2 |  |
| 1 (slope 1: 0-3) | 3598.23 (10) |  | 0.53 | 0.00 | 0.61 | 34.54 |  | -5.81 |  | 0.180 |  |  |  |  |  |
| **2 (slope 1: 0-12)** | **3512.69 (10)** |  | **0.53** | **0.00** | **0.61** | **34.08** |  | **-1.35** |  | **0.741** |  |  |  |  |  |
| 3 (slope 1: 0-12) | 2758.75 (9) | 753.94 (1)*** | 0.49 | 0.29 | 0.99 | 34.06 |  | -1.35 | 18.75 | 0.741 |  |  |  |  |  |
| 4 (slope 1: 0-12) | 2360.54 (7) | 398.21 (2)*** | 0.52 | 0.21 | 0.61 | 34.06 |  | -1.35 | 13.47 | 0.74 | 6.71 |  |  | 0.08 |  |
| 5 (slope 1: 0-12) | 374.69 (4) | 1985.85 (3)*** | 0.27 | 0.86 | 0.04 | 34.06 | 24.97 | -1.35 | 3.80 | 0.741 | 0.67 | -0.95* | 0.20 | 0.08 |  |
| Conditional | 545.51 (16) | NA | 0.16 | 0.90 | 0.02 | 36.01 | 24.16 | 2.07 | 3.46 | 0.83 | 0.61 | -0.034** | 0.20* | 0.15 |  |

Table 12. Piecewise latent growth curve analysis of habit strength

| Model | Fit statistics | | | | | Intercept | | Slope | | Slope 2 | | Covariance | | | |
| --- | --- | --- | --- | --- | --- | --- | --- | --- | --- | --- | --- | --- | --- | --- | --- |
|  | Chi-Square (df) | Change in Chi-Square (df) | RMSEA | CFI | SRMR | mean | variance | mean | variance | mean | variance | Intercept, slope | Intercept, slope 2 | Slope 2, slope 2 |  |
| 1 (slope 1: 0-3) | 1008.93 (10) |  | 0.28 | 0.00 | 0.36 | 3.26 |  | 2.97 |  | -0.11 |  |  |  |  |  |
| 2 (slope 1: 0-12) | 1107.99 (10) |  | 0.29 | 0.00 | 0.36 | 3.59 |  | 0.45 |  | -0.18 |  |  |  |  |  |
| 3 (slope 1: 0-3) | 810.01 (9) | 397.98 (1)*** | 0.21 | 0.38 | 0.34 | 3.26 |  | 297 | 12.64 | -0.11 |  |  |  |  |  |
| 4 (slope 1: 0-3) | Not converged | |  |  |  |  |  |  |  |  |  |  |  |  |  |
| 5 (slope 1: 0-3) | Not converged | |  |  |  |  |  |  |  |  |  |  |  |  |  |
| Conditional | 484.79 (15) | NA | 0.16 | 0.57 | 0.21 | 1.94 |  | 2.47 | 11.67 | -0.01 |  |  |  |  |  |

Table 13. Latent growth curve analysis of dietary restraint

| Model | Fit statistics | | | | | Intercept | | Slope | | Slope 2 | | Covariance | | | |
| --- | --- | --- | --- | --- | --- | --- | --- | --- | --- | --- | --- | --- | --- | --- | --- |
|  | Chi-Square (df) | Change in Chi-Square (df) | RMSEA | CFI | SRMR | mean | variance | mean | variance | mean | variance | Intercept, slope | Intercept, slope 2 | Slope 2, slope 2 |  |
| 1 (slope 1: 0-3) | 740.49 (10) |  | 0.24 | 0.00 | 0.32 | 5.15 |  | 10.30 |  | -0.67 |  |  |  |  |  |
| 2 (slope 1: 0-12) | 944.33 (10) |  | 0.27 | 0.00 | 0.38 | 6.30 |  | 1.42 |  | -0.97 |  |  |  |  |  |
| 3 (slope 1: 0-3) | 325.35 (9) | 618.98 (1)*** | 0.17 | 0.44 | 0.26 | 5.15 |  | 10.30 | 54.16 | -0.67 |  |  |  |  |  |
| 4 (slope 1: 0-3) | Not converged | |  |  |  |  |  |  |  |  |  |  |  |  |  |
| 5 (slope 1: 0-3) | Not converged | |  |  |  |  |  |  |  |  |  |  |  |  |  |
| Conditional | 319.61 (15) | NA | 0.13 | 0.65 | 0.16 | 2.85 |  | 8.12 | 49.63 | -0.37 |  |  |  |  |  |

Table 14. Latent growth curve analysis of autonomous diet self-regulation

| Model | Fit statistics | | | | | Intercept | | Slope | | Slope 2 | | Covariance | | | |
| --- | --- | --- | --- | --- | --- | --- | --- | --- | --- | --- | --- | --- | --- | --- | --- |
|  | Chi-Square (df) | Change in Chi-Square (df) | RMSEA | CFI | SRMR | mean | variance | mean | variance | mean | variance | Intercept, slope | Intercept, slope 2 | Slope 2, slope 2 |  |
| 1 (slope 1: 0-3) | 952.34 (10) |  | 0.27 | 0.00 | 0.49 | 6.00 |  | -0.58 |  | -0.20 |  |  |  |  |  |
| 2 (slope 1: 0-12) | 954.46 (10) |  | 0.27 | 0.00 | 0.47 | 5.98 |  | -0.32 |  | -0.15 |  |  |  |  |  |
| 3 (slope 1: 0-3) | Not converged | |  |  |  |  |  |  |  |  |  |  |  |  |  |
| 4 (slope 1: 0-3) | Not converged | |  |  |  |  |  |  |  |  |  |  |  |  |  |
| 5 (slope 1: 0-3) | Not converged | |  |  |  |  |  |  |  |  |  |  |  |  |  |
| Conditional | 319.61 (15) | NA | 0.20 | 0.37 | 0.39 | 2.85 |  | 8.12 | 49.63 | -0.37 |  |  |  |  |  |

**Table 15.** *Associations between the latent growth factors of BMI and potential mediators*

| **Variable** | **BMI growth factors** | | **Fit statistics** | | |
| --- | --- | --- | --- | --- | --- |
|  | **Slope** | **Slope 2** | **CFI** | **RMSEA** | **SRMR** |
| Dietary Restraint | | | | | |
| Slope | -0.45 (0.13) ** | -0.51 (2.54) | 0.72 | 0.14 | 0.12 |
| Slope 2 |  | -1.70 (5.84) |  |  |  |
| Habit strength | | | | | |
| Intercept | -0.61 (0.19)** | 0.26 (0.14) | 0.76 | 0.14 | 0.12 |
| Slope |  | -0.56 (0.15)** |  |  |  |
| Slope 2 |  |  |  |  |  |
| Autonomous diet Self-regulation | | | | | |
| Slope | -1.17 (0.50)* | 0.74 (0.30)* | 0.61 | 0.13 | 0.17 |
| Slope 2 |  | 1.63 (1.25) |  |  |  |

*Note.* * *p* < .05. ** *p* < .01. *** *p* < .001. BMI, Body Mass Index. CFI, comparative fit index. RMSEA, Root mean square error of approximation. SRMR, Standardized Root Mean Square Residual.


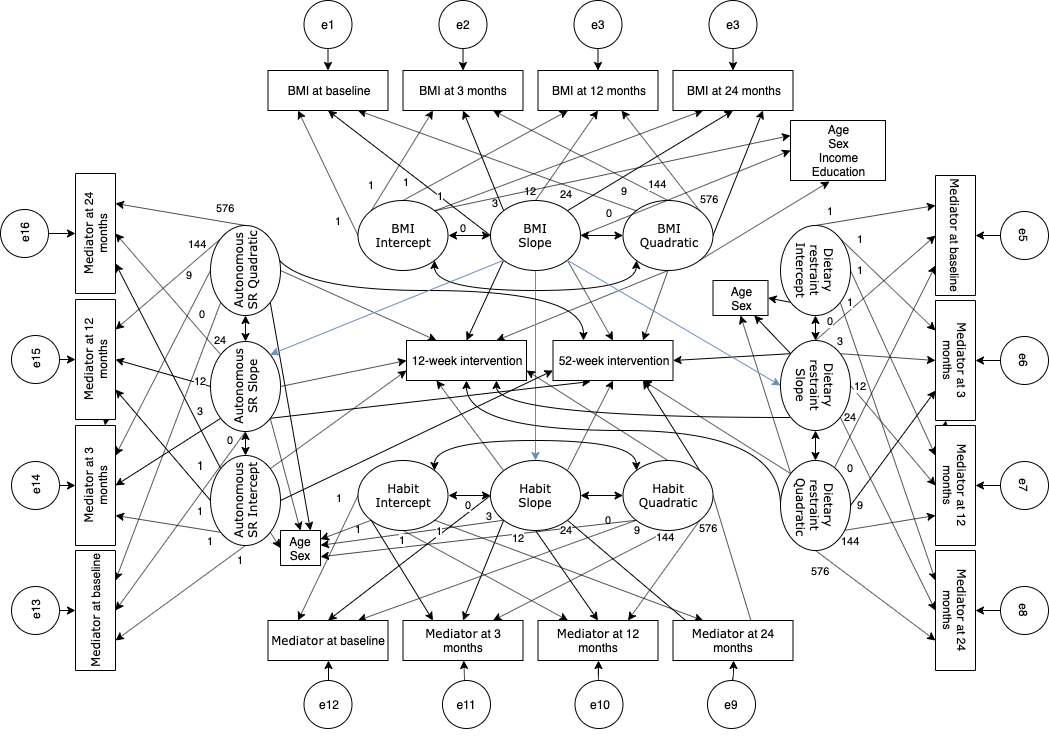


Figure 6. Full mediation model tested

Table 16. Total, direct and indirect effects via mediating variables of the 12- and 52-week intervention on BMI for each mediator (tested in separate models)

| **Effect** | Total impact of intervention on BMI | | | Total indirect effect of mediating variables | | | Direct effect of intervention on BMI when mediator included | | | |
| --- | --- | --- | --- | --- | --- | --- | --- | --- | --- | --- |
|  | **Estimate** | **SE** | **P value** | **Estimate** | **SE** | **P value** | **Estimate** | **SE** | **P value** |  |
| Habit strength | | | | | | | | | |  |
| 12-week intervention | -0.68 | 0.37 | 0.06 | -0.599 | 0.30 | 0.04 | -0.08 | 0.45 | 0.86 |  |
| 52-week intervention | -1.72 | 0.38 | <0.001 | -1.08 | 0.39 | 0.01 | -0.65 | 0.53 | 0.22 |  |
| Dietary restraint | | | | | | | | | |  |
| 12-week intervention | -0.68 | 0.37 | 0.06 | -0.83 | 0.34 | 0.014 | 0.14 | 0.51 | 0.78 |  |
| 52-week intervention | -1.72 | 0.38 | <0.001 | -1.41 | 0.47 | 0.003 | -0.31 | 0.60 | 0.60 |  |
| Autonomous diet self-regulation | | | | | | | | | |  |
| 12-week intervention | -0.68 | 0.37 | 0.06 | -0.21 | 0.17 | 0.21 | -0.442 | 0.39 | 0.26 |  |
| 52-week intervention | -1.72 | 0.38 | <0.001 | -0.39 | 0.18 | 0.03 | -1.32 | 0.40 | 0.001 |  |

Table 17. Standardised total, direct and indirect effects via mediating variables of the 12- and 52-week intervention on BMI

| **Effect** | **12-week intervention** | | | **52-week intervention** | | | | | |
| --- | --- | --- | --- | --- | --- | --- | --- | --- | --- |
|  | **Estimate** | **SE** | **P value** | **Estimate** | | **SE** | | **P value** | |
| Total impact of intervention on BMI | -0.15 | 0.06 | 0.01 | -0.28 | | 0.06 | | <0.001 | |
| Total indirect effect of mediating variables | -0.17 | 0.06 | 0.002 | -0.29 | | 0.07 | | <0.001 | |
| Direct effect of intervention on BMI when mediators included | 0.02 | 0.08 | 0.78 | 0.002 | | 0.10 | | 0.983 | |
| *Indirect Effect of Mediator* | | | | | | | | | |
| Habit | -0.05 | 0.03 | 0.07 | | -0.09 | | 0.04 | | 0.02 |
| Restraint | -0.10 | 0.04 | 0.027 | | -0.16 | | 0.06 | | 0.009 |
| DSR | -0.02 | 0.02 | 0.154 | | -0.04 | | 0.02 | | 0.048 |

**References**

Curran, P. J., Obeidat, K., & Losardo, D. (2010). Twelve frequently asked questions about growth curve modeling. *Journal of cognition and development*, *11*(2), 121-136.

Hamilton, J., Gagne, P. E., & Hancock, G. R. (2003). The Effect of Sample Size on Latent Growth Models.

Hertzog, C., Lindenberger, U., Ghisletta, P., & von Oertzen, T. (2006). On the power of multivariate latent growth curve models to detect correlated change. *Psychological methods*, *11*(3), 244.

Kamata, A., Nese, J. F., Patarapichayatham, C., & Lai, C.-F. (2013). Modeling nonlinear growth with three data points: Illustration with benchmarking data. *Assessment for Effective Intervention*, *38*(2), 105-116.

MacCallum, R. C., Kim, C., Malarkey, W. B., & Kiecolt-Glaser, J. K. (1997). Studying multivariate change using multilevel models and latent curve models. *Multivariate Behavioral Research*, *32*(3), 215-253.

Preacher, K. J., Wichman, A. L., MacCallum, R. C., & Briggs, N. E. (2008). *Latent growth curve modeling*. Sage.

Singer, J. D., & Willett, J. B. (2003). *Applied longitudinal data analysis: Modeling change and event occurrence*. Oxford university press.

Van de Schoot, R., Lugtig, P., & Hox, J. (2012). A checklist for testing measurement invariance. *European Journal of Developmental Psychology*, *9*(4), 486-492.

Wickrama, K. K., Lee, T. K., O’Neal, C. W., & Lorenz, F. O. (2016). *Higher-order growth curves and mixture modeling with Mplus: A practical guide*. Routledge.
